# Molecular Networking-Based Analysis of Cytotoxic Saponins from Sea Cucumber *Holothuria atra*

**DOI:** 10.3390/md17020086

**Published:** 2019-02-01

**Authors:** Laura Grauso, Afsaneh Yegdaneh, Mohsen Sharifi, Alfonso Mangoni, Behzad Zolfaghari, Virginia Lanzotti

**Affiliations:** 1 Dipartimento di Agraria, Università di Napoli Federico II, Via Università 100, 80055 Portici (NA), Italy; laura.grauso@unina.it; 2 Department of Pharmacognosy, School of Pharmacy, Isfahan University of Medical Sciences, Hezar-Jerib Ave., 81746 73461 Isfahan, Iran; yekdaneh@pharm.mui.ac.ir (A.Y.); shmohsen133@gmail.com (M.S.); behzadz@gmail.com (B.Z.); 3 Dipartimento di Farmacia, Università di Napoli Federico II, Via Domenico Montesano 49, 80131 Napoli, Italy; mangoni@unina.it

**Keywords:** sea cucumbers, holothurians, saponins, triterpene glycosides, LC-MS/MS, molecular networking, cytotoxic activity

## Abstract

The saponin composition of a specimen of black sea cucumber, *Holothuria atra* collected in the Persian Gulf was studied by a combined approach including LC-MS/MS, Molecular Networking, pure compound isolation, and NMR spectroscopy. The saponin composition of *Holothuria atra* turned out to be more complex than previously reported. The most abundant saponins in the extract (**1**–**4**) were isolated and characterized by 1D- and 2D-NMR experiments. Compound **1** was identified as a new triterpene glycoside saponin, holothurin A5. The side chain of the new saponin **1**, unprecedented among triterpene glycosides, is characterized by an electrophilic enone function, which can undergo slow water or methanol addition under neutral conditions. The cytotoxic activity of compounds **1**–**4**, evaluated on the human cervix carcinoma HeLa cell line, was remarkable, with IC_50_ values ranging from 1.2 to 2.5 µg/mL.

## 1. Introduction

Holothurians are marine echinoderms commonly referred to as sea cucumbers. Over 1400 species of holothurians have been described and they are found all around the world [1]. Holothurians have been attracting increasing interest for their high content of secondary metabolites with manifold potential applications, especially in the pharmaceutical field [2]. The biological potential of sea cucumbers ranges from antitumor to antimicrobial, anti-inflammatory and antiviral activities [3,4,5,6,7,8]. Moreover, it has been reported that sea cucumbers extracts may act as anticoagulant agents and inhibitors of hyperlipidemia [9,10]. In addition to their pharmacological activities, secondary metabolites from holothurians are also attractive for their possible applications in the nutraceutical and cosmeceutical fields [11,12,13]. 

Their use in nutraceutical industry appears particularly appealing because some species of holothurians are used as food in several regions of Asia, and therefore are known to be devoid of toxicity. The growing demand of edible sea cucumbers is linked to their supposed benefits to human health on account of their high content of nutrients, including vitamins and metals. Additionally, they are the consumed because they are believed to be an aphrodisiac food [13]. Although many different classes of secondary metabolites have been isolated from sea cucumbers, saponins, and in particular triterpene glycosides, are their signature metabolites. Saponins from holothurians are typically composed of an aglycone based on the lanostane skeleton and an oligosaccharide chain. The number of monosaccharides in the sugar chain ranges from 1 to 6, and one or more of them can bear a sulfate group [14]. The biological activity of saponins is generally attributed to their strong membranolytic action following their interaction with sterols of cellular membrane [15]. Saponins from holothurians are believed to have an ecological role as defense compounds against predators [16], although they can also play an important role in attracting symbionts [17].

Black sea cucumber, *Holothuria atra* is typically found in the Pacific and Indian Oceans. It is an edible sea cucumber with a high content of bioactive compounds. To date, many papers have been published showing that the extracts of *H. atra* possess a wide range of different biological activities. However, only a few studies on the chemical profile, and more specifically on the saponin profile of *H. atra* have been performed, and they are limited to specimens collected off the Madagascar coasts [18,19] and off the Island of Okinawa [14].

**Figure 1:**
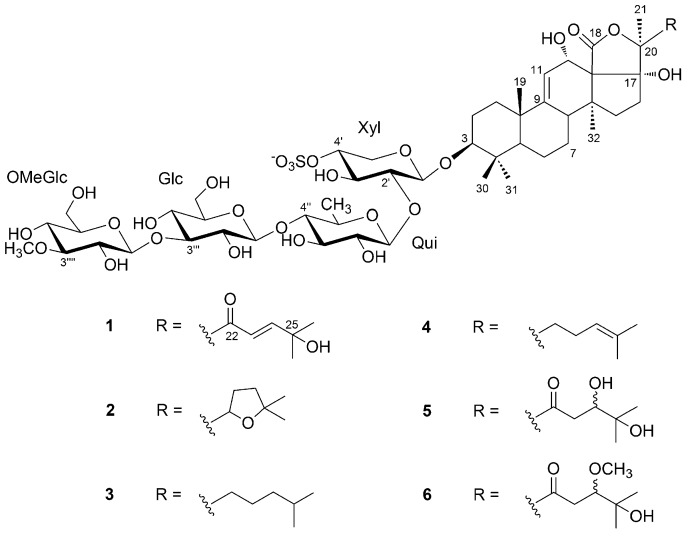
Major holothurins from black sea cucumber *Holothuria atra* collected in Persian Gulf.

In this study, we report on the saponin composition of a specimen of *H. atra* collected in a different geographic area, namely the Persian Gulf, using a modern analytical approach that combines tandem mass spectrometry (MS/MS) and molecular networking, a recent computational technique for the automatic identification of structurally related molecules on the basis of their fragments in the MS/MS spectrum [20]. The most abundant saponins identified were isolated, and their structures determined by NMR methods, affording the new saponin holothurin A5 (**1**), and confirming the structure of three known saponins (**2**–**4**) (Figure 1). Preliminary evaluation of the cytotoxic activity of **1**–**4** is also reported.

## 2. Results

### 2.1. Saponin Profile and MS/MS-Based Molecular Networking

A specimen of *H. atra* was collected in the Persian Gulf, Iran, in October 2015 through the Kupchan partitioning method [21] (see the Materials and Methods section for details). 

**Figure 2:**
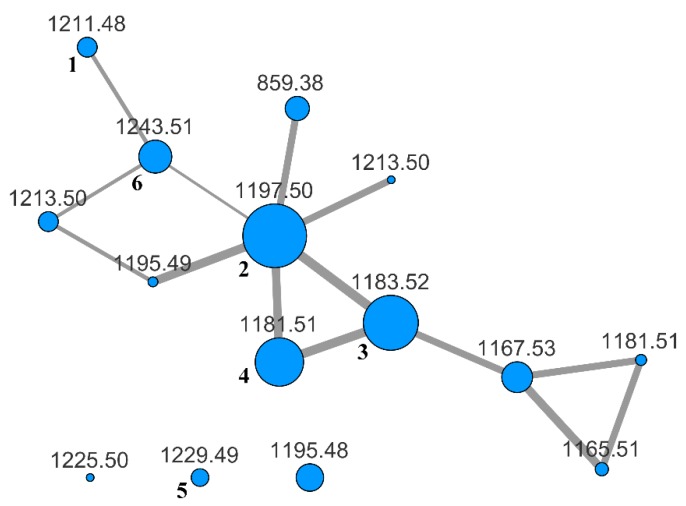
MS/MS-based molecular network of the BuOH extract of *H. atra*. Each node is labeled with the parent mass. Node size is relative to ion count, edge thickness is relative to cosine score. Nodes with the same parent mass refer to isomeric compounds. They were automatically recognized by the MS-Cluster module of GNPS based on their different MS/MS spectra.

To evaluate the whole content of saponins, the BuOH extract was subjected to LC-MS and LC-MS/MS analysis on an LTQ-Orbitrap instrument using the pentafluorophenyl (PFP) stationary phase and a MeOH/water gradient program. The analysis was performed in the negative-ion mode, because it is known that almost all the saponins from *H. atra* contain a sulfated sugar and are therefore anionic in solution. Fragmentation of saponins, obtained using collision-induced dissociation (CID), produced few fragment ions using the default parameters. Hence, the fragmentation was enhanced by turning on the wideband activation option and increasing the activation time up to 80 ms. In addition, we found that a better network was obtained when the isotope peaks were removed from the MS/MS spectra with the msconvert program [22] during the pre-processing of raw data obtained from the LC-MS/MS run. Finally, the pre-processed file was uploaded to the online platform at Global Natural Products Social Molecular Networking website (gnps.ucsd.edu), and a molecular network was built using the small dataset workflow. The resulting network was visualized using the Cytoscape program [23] and is shown in Figure 2.

The specimen of *H. atra* under investigation showed a moderately complex saponin profile. Many nodes in the networks were relative to molecular weights that fit metabolites previously isolated from *H. atra* or other holothurians (Table 1), but several nodes had no obvious association with known metabolites.

Therefore, the extract was subjected to HPLC chromatography in the attempt to isolate pure saponins and determine their structures. Owing to the difficulties to scale-up the LC-MS method to a preparative scale for compounds generally devoid of chromophores, we could only isolate in the pure form the four most abundant saponins **1**–**4**. Compounds **2**–**4** were already known saponins, and were identified as holothurin A (**2**) [24], echinoside A (**3**) [25], and 24-dehydroechinoside A (**4**) [26] on the basis of their NMR spectra by comparison with literature data. In addition, their ^1^H and ^13^C NMR spectra were also recorded in CD_3_OD solution (for consistency with the studies performed on compound **1** in the same solvent) and fully assigned (Tables S1–S3 in the Supplementary Materials section). Compound **1** is a new saponin, which we called holothurin A5, and its structure elucidation is described in Section 2.2 below. Compounds **5** and **6** were also isolated in the pure form, but as mixtures of epimers. They are also new saponins, but were shown to be likely artifacts (see Section 2.3 below).

### 2.2. Structural Determination of Holothurin A5 (**1**) 

The molecular formula of holothurin A5 (**1**) was established as C_54_H_83_O_28_S^−^ from the M^−^ ion at 1211.4809 in the negative-ion mode high-resolution ESI-MS spectrum, as well as by the molecular ion [M + 2Na]^+^ at 1257.4600 in the positive-ion mode spectrum. 

Preliminary analysis of the 1D ^1^H- and ^13^C-NMR spectra (Figure S1 in the Supplementary Materials section) showed the presence of seven methyl groups, two carbon-carbon double bonds (δ_H_ 5.39, δ_C_ 115.1 and 157.2, δ_H_ 6.85 and 7.14, δ_C_ 121.3 and 157.6), a lactone carbonyl carbon (δ_C_ 175.0), a ketone carbonyl carbon (δ_C_ 198.0), an oxymethine group (δ_H_ 4.55, δ_C_ 71.8), a non-protonated carbinol (δ_C_ 71.9), and signals belonging to an oligosaccharide chain (δ_H_ 3.0–4.65) (Table 2). The combined MS and NMR data suggested **1** to be a monosulfated triterpene glycoside.

The oligosaccharide chain was composed of four monosaccharide residues, as indicated by the four anomeric protons and carbons displayed by the HSQC 2D NMR spectrum. The coupling constants of the anomeric protons, in the range 7.1–7.9 Hz, indicated the β configuration of all the four sugar residues. Combined interpretation of 2D-COSY, *z*-TOCSY, and HSQC spectra allowed identification of the structure of each monosaccharide, which was connected by the presence of HMBC correlations between protons and carbons belonging to different sugars (Figures S2–S5).

The spin system of the first sugar residue, identified by the z-TOCSY experiment, belonged to a pentose, in that it contained four methine and one methylene groups (Table 2). A strong HMBC correlation between H-5’ and C-1’ showed the pentose to be in the pyranose form. Moreover, the de-shielded chemical shift of H-4’ (δ 4.22) indicated that the sugar was sulfated at O-4′. Proton-proton coupling constants indicated that H-2’, H-3’ and H-4’ were axial, and therefore that the first sugar residue was identified as a xylose 4-O-sulfate (Xyl). 

**Figure 3:**
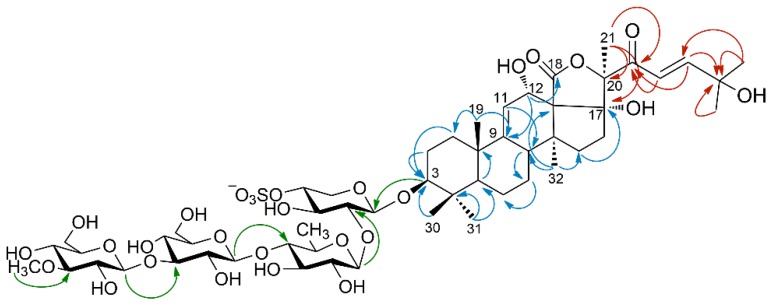
Diagnostic HMBC correlations for the oligosaccharide chain (green arrows), the holostane nucleus (blue arrows), and the side chain (red arrows) of compound **1**.

Likewise, the other three sugar residues were identified as a quinovose residue (Qui) and two glucose residues, all in their pyranose form. The HMBC correlation of the methoxy protons at δ 3.64 with C-3′′′′ and, conversely, the coupling of H-3′′′′ with the methoxy carbon atom showed that one glucose residue was methylated at O-3 (OMeGlc). The monosaccharides sequence was determined by diagnostic HMBC cross peaks between H-1′ (Xyl) and C-3 of the holostane, H-1′′ (Qui) and C-2′ (Xyl), H-1′′′ (Glc) and C-4′′ (Qui), and H-1′′′′ (OMeGlc) and C-3′′′ (Glc) (Figure 3), and turned out to be the same sugar sequence as in compounds **2**–**4**.

An extensive analysis of 2D-NMR, and in particular the overall consideration of the correlations peaks in the HMBC spectrum (Figure 3), determined the structure of the aglycone. It was based on the typical holostane skeleton, i.e., a 5-α-lanostane skeleton with a lactone ring closed between the oxidized C-18 and C-20. The holostane showed additional functions, i.e., a Δ9 unsaturation and hydroxyl groups at C-3, C-12, and C-17. The pentacyclic nucleus of **1** is therefore identical to that of compounds **2**–**4**. The nearly complete coincidence of the ^1^H and ^13^C chemical shifts of the nucleus of **1** with those of **2**–**4** showed that also the stereochemistry of the nucleus was the same. 

The unprecedented side chain of **1** required de novo structure elucidation. The presence of an unsaturated ketone was suggested by the UV absorption at 234 nm, and confirmed by the presence of two mutually coupled doublets at δ 7.14 and 6.85 in the ^1^H-NMR spectrum. The coupling constant of these signals (15.5 Hz) indicated the trans-configuration of the double bond. The HMBC cross-peaks shown in Figure 3 located the α,β-unsaturated ketone in the side chain, and an additional hydroxyl group on the non-protonated C-25 carbon based on its chemical shift.

Holothurin A5 (**1**) shares the oligosaccharide chain and the holostane skeleton with many other triterpene glycosides, previously reported in literature, but possesses an unprecedented side chain. The most similar known saponin is holothurin A3, whose side chain shows a carbonyl group at position 22 and an hydroxyl group at position 25 [27]. 

### 2.3. Solvent Addition to Holothurin A5 (**1**) and Structure of Compounds **5** and **6**

**Figure 4:**
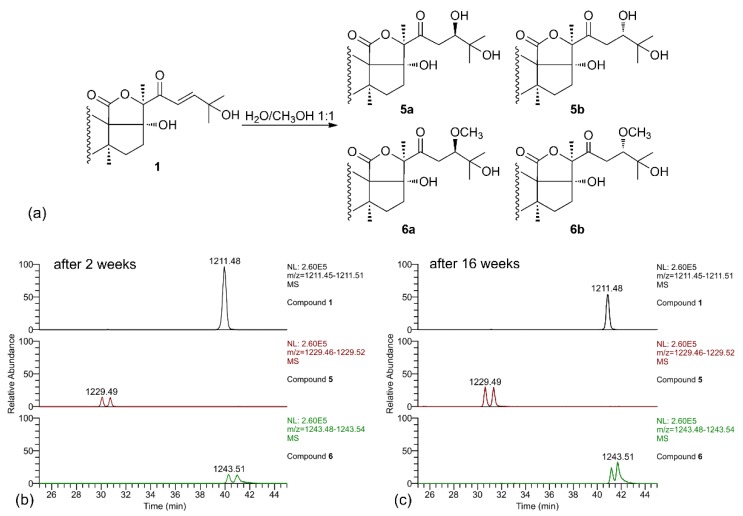
(**a**) Scheme of the solvolysis reaction occurring between the α,β-unsaturated ketone in the side chain of holothurin A5 (**1**) and a solution of MeOH/H_2_O (1:1); (**b**) LC-MS analysis of compound **1** after having left the compound in a solution of MeOH/H_2_O (1:1). Two isomeric saponins at 1229.5 (addition of H_2_O) and two isomeric saponins at 1243.5 (addition of MeOH) appeared in the spectrum; (**c**) LC-MS analysis of compound **1** left in the same solution of MeOH/H_2_O (1:1) repeated after 16 weeks. The peaks of the additional saponins showed an increasing intensity.

When the LC-MS analysis of a pure sample of compound **1** was repeated after leaving it in MeOH/water solution for two weeks, new peaks were present in the chromatograms, corresponding to compounds **5** and **6** (each present as an isomeric pair). Their molecular formulas (C_54_H_85_O_29_S^−^ for **5** and C_55_H_87_O_29_S^−^ for **6**), contained, respectively, one additional water molecule and one additional MeOH molecule compared to **1**, suggesting that they could be derived from compound **1** by addition of the solvent. A further confirm of this hypothesis was obtained when the LC-MS analysis was repeated again after 16 weeks, and a remarkable increase of the amounts of compounds **5** and **6** was observed (Figure 4). Therefore, compounds **5** and **6** were isolated (no attempt was made to separate the stereoisomers), and their structure elucidated by NMR.

Analysis of the 2D-NMR spectra of **5** showed that signals of the holostane nucleus and of the oligosaccharide chain were coincident with those of **1**, and that the difference was confined in the side chain. In particular, the de-shielded olefinic protons H-23 and H-24 were not present to the ^1^H NMR spectrum, while a new oxymethine proton (H-24, δ 3.95) appeared in the spectrum (Figure S6). A new, quite deshielded methylene group (H_2_-23, δ 2.83 and 2.87 for the two isomers), coupled with the oxymethine H-24, was also present in the spectrum. These data strongly suggested that **5** is the hydrated derivative of compound **1** at the Δ23 double bond, and that the two isomers of **5** are the two possible epimers at C-24. This hypothesis was confirmed by the HMBC spectrum, which showed clear correlation peaks between the methyl protons H_3_-26 and H_3_-27 and the oxymethine carbon C-24, and between H-24 and the ketone carbonyl group C-22, and allowed the full assignment of the side chain signals for the two epimers (Table 3). Likewise, the ^1^H NMR spectra of compound **6** did not contain the Δ24 double bond and the signals of a –CH_2_CH(OCH_3_)– system at positions 23 and 24 (Figure S7). The HMBC spectrum confirmed this structural assignment with similar correlation peaks as those described for **5**, with the additional correlation of the methoxy protons with C-24. 

It is interesting to note that compounds with the same mass as **5** and **6** were present in the molecular network, and a retrospective analysis of the LC-MS data showed that also their retention times are the same. Likely, compound **6** was formed during the extraction process, in which MeOH is used; as for compound **5**, this could be formed from **1** during the extraction as well, but also in the living sea cucumber, and therefore may have some physiological significance.

### 2.4. Biological Activity of Compounds **1**–**4**

The cytotoxic activity of the pure compounds **1**–**4** against HeLa tumor cell-lines was assessed by 3-(4,5-dimethylthiazol-2-yl)-2,5-diphenyl tetrazolium bromide (MTT) assay. The activity was measured based on cell viability after 72 h of treatment. Compounds **1**–**4** showed cytotoxic activity against the human cervix carcinoma HeLa cell-line, with IC_50_ in the range 1.2–2.5 µg/mL (Table 4). Among them, compound **3** was the most potent, with IC_50_ of 1.2 µg/mL. Compounds **1**–**4** were almost as potent as the positive control adriamycin (IC_50_ of 0.6 µg/mL), an antitumor drug in clinical use, as for toxicity to HeLa cells.

## 3. Discussion

Sea cucumbers were shown, once again, to be an important source of bioactive compounds. The analyzed specimen of *H. atra* was shown to contain a number of triterpene glycosides, some of which unprecedented. Holothurin A (2) and echinoside A (also known as holothurin A2, 3) are two of the most widespread holothurins, and have been previously reported from *H. atra*. In contrast, 24-dehydroechinoside A (4), quite common in other species of *Holothuria*, is reported here for the first time from *H. atra*. The new saponin holothurin A5 (1) shows a remarkable structural interest because of its side chain, which has been never reported in any terpene glycosides from any organism. Some examples of a similar side chain, but not involved in a lactone ring, have been found in some triterpenoid glycosides from Cucurbitaceae [28,29] and, in the marine environment, in a single steroid glycoside from a starfish [30].

Holothurin A5 (**1**) is able to undergo nucleophilic addition by the solvent under in neutral conditions, and this ability is clearly linked to the α,β-unsaturated ketone function in its side chain. The strong electrophilic character of **1** could cause it to react with a large number of nucleophilic targets in the intracellular or intercellular environment, and this could significantly affect the biological activity of compound **1** compared to the other saponins present in *H. atra*. In spite of this, in the preliminary cytotoxicity assay performed on HeLa cell lines, compound **1** did not show any remarkably higher activity, and all the saponins tested were active at similar concentrations (few μg/mL) in this specific assay. Nevertheless, the electrophilicity of the side chain of **1** could modulate the bioactivity of the saponin in more subtle ways, not exploited in a simple cytotoxicity assay. This possibility will be developed in the future.

**Figure 5:**
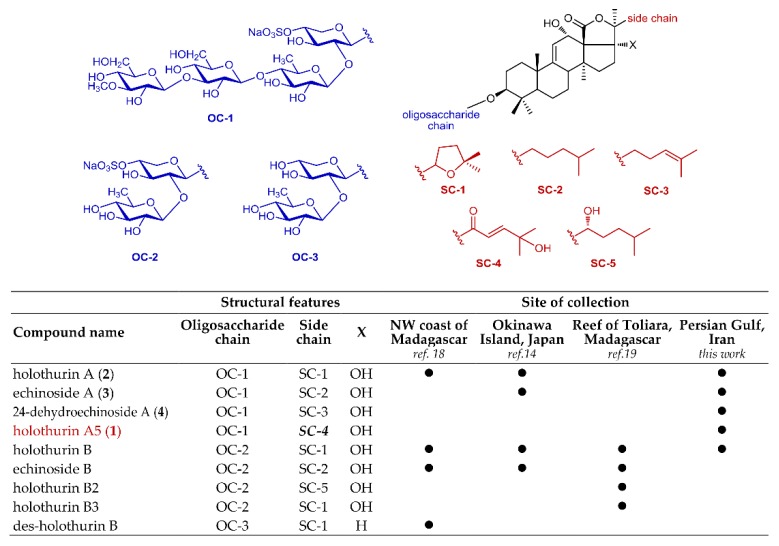
A comparison of major triterpene glycosides found in the specimens of *Holothuria atra* studied so far.

Another interesting consideration regards the saponin content of *H. atra* and how the results of our analysis fit previous analyses of *H. atra*, which were performed on sample collected in different areas [14,18,19]. Previous papers on the saponin profile of *H. atra* showed the presence of two main groups of saponins, those with four sugars in the sugar chain (e.g., holothurin A) and those with two sugars in the chain (e.g., holothurin B). While the saponins with two sugar are predominant, or even exclusive, in other specimens, we only found saponins with four sugars, except for very small amounts of holothurin B (Figure 5). While the geographical origin of the specimens is undoubtedly very important, some of the differences could also be ascribed to differences between individual specimens and to the different analytical methods used in different laboratories. For example, in early works [14] reflux extraction was a common practice, and it was conceivable that saponins with two sugars could be formed by partial hydrolysis under these harsh conditions; however, a more recent paper [19] that reports only on saponins with two sugars from *H. atra* from Toliara, extraction was made under carefully controlled conditions. Therefore, the artifactual nature of saponins with two sugars seems to be unlikely. In addition, in the latter study [19], analysis was performed on several specimens of *H. atra*, and no saponins with four sugars was found in any of them; therefore, even though we had the chance to analyze only one specimen of *H. atra* from Persian Gulf and our results should only be considered as indicative, the different composition is clearly linked to the geographical origin. In our opinion, an unbiased and isolation-independent analytical method like LC-MS/MS combined with molecular networking could be the basis for future, more meaningful, comparisons. 

## 4. Materials and Methods 

### 4.1. General Experimental Procedures

NMR spectra were determined on Varian Unity Inova spectrometers at 700 MHz and 500 MHz. Chemical shifts were referenced to the residual solvent signal (CD_3_OD: δ_H_ 3.31, δ_C_ 49.0). For an accurate measurement of the coupling constants, the one-dimensional ^1^H NMR spectra were transformed at 128 K points (digital resolution < 0.1 Hz). The HSQC spectra were optimized for ^1^*J*_CH_ = 142 Hz and the ^13^C HMBC experiments for ^2,3^*J*_CH_ = 8.0 Hz. Medium pressure liquid chromatography were performed on a Buchi Gradient System C-605 apparatus using glass columns of LiChroprep RP-18 (25–40µm) and a C-660 Buchi fraction collector. TLC performed on SiO_2_ plates with BuOH/H_2_O/CH_3_CO_2_H 60:25:15 (BAW) as a mobile phase and cerium sulphate in 2 N H_2_SO_4_ as a reagent for visualizing the spots. High performance liquid chromatography (HPLC) separations were achieved on a Waters 515 apparatus equipped with a refractive index detector (Waters 2414) using semi-preparative Novapak C18 (300 mm × 7.8 mm i.d.) and analytical Novapak C18 (300 mm × 3.9 mm i.d.) columns. Final purification were performed on an Agilent 1260 Infinity Quaternary LC apparatus (Agilent Technology, Cernusco sul Naviglio, Italy) equipped with a Diode-Array Detector (DAD). 

### 4.2. Sample Collection, Extraction, and Isolation

*Holothuria atra* was collected at Persian Gulf, Iran in October 2015. Identification of the sea cucumber was kindly carried out by Seyed Mohammad Bagher Nabavi from Khoramshahr Marine Science and Technology University. The animal (1.4 kg wet weight) was cut into pieces, dried by freeze drier and extracted four times with EtOAc/MeOH (1:1). The solvent was evaporated by rotary evaporator. Desired extract was partitioned by Kupchan partitioning method to hexane, dichloromethane, butanol and water [21]. Butanol partition was further fractionated by MPLC; RP18 was used as stationary phase and column was eluted with a gradient solvent system from 100% H_2_O to pure MeOH. Some fractions containing saponin compounds were finally purified to yield compounds **1**–**6**.

The final purification of compounds **1**, **5** and **6** was performed on a HPLC column Luna C-18 (250 mm × 4.60 mm) with a gradient of elution of H_2_O (A) and MeOH (B), 40–80% B over 30 min, 80%–100% B over 5 min, and hold 10 min, flow rate 0.8 mL/min (**1**, t_R_ = 15.6 min; **5**, t_R_ =10.6 min; **6**, t_R_ = 16.2 min).

Holothurin A5 (**1**): colorless amorphous solid. HRESIMS (positive ion mode) *m*/*z* 1257.4600 ([M + 2Na]^+^, *m*/*z* 1221.4879 ([M + Mg]^+^, *m*/*z* 1237.4652 ([M + Ca]^+^; HRESIMS (negative ion mode) *m*/*z* 1211.4809 (calcd. for C_54_H_84_O_28_S *m*/*z* 1211.4797); ^1^H and ^13^C NMR: Table 2; UV (MeOH) λ_max_ (ε): 234 nm (11,000). 

Compound **5**: colorless amorphous solid. HRESIMS (positive ion mode) *m*/*z* 1275.4706 ([M + 2Na]^+^, *m/z* 1253.4760 ([M + Mg]^+^, *m*/*z* 1269.4537 ([M + Ca]^+^; HRESIMS (negative ion mode) *m*/*z* 1229.4912 (calcd. for C_54_H_86_O_29_S *m*/*z* 1229.4903); ^1^H and ^13^C NMR: Table 3. 

Compound **6**: colorless amorphous solid. HRESIMS (positive ion mode) *m*/*z* 1289.4863 ([M + 2Na]^+^, *m/z* 1267.4919 ([M + Mg]^+^, *m*/*z* 1283.4693 ([M + Ca]^+^; HRESIMS (negative ion mode) *m*/*z* 1243.5064 (calcd. for C_55_H_87_O_29_S *m*/*z* 1243.5059); ^1^H and ^13^C NMR: Table 3. 

### 4.3. LC-HRMS and LC-HRMS/MS

All LC-MS and LC-MS/MS experiments were performed on a Thermo LTQ Orbitrap XL mass spectrometer (Thermo Fisher Scientific Spa, Rodano, Italy) coupled to a Thermo U3000 HPLC system (Agilent Technology, Cernusco sul Naviglio, Italy). The LC-MS method was created on a Kinetex 5 µm, 100 mm × 2.1 mm PFP column (Phenomenex, Torrance, CA, USA), with 0.1% formic acid in H_2_O as solvent A and CH_3_OH as solvent B. The gradient elution was optimized as follows: 40% B 3 min, 40% to 100% B over 20 min, hold 10 min, flow rate 0.2 mL/min. Injection volume was 5 μL. 

Preliminary high-resolution mass spectra (HR-MS) and high-resolution tandem mass spectra (HR-MS/MS) were acquired either in positive than in negative ion detection mode in the range of *m*/*z* 200–1800 with resolution set to 60,000. Based on the preliminary results, the subsequent analyses were performed in the negative-ion mode with a mass range of 700–1800 for MS scans (mass range for each MS/MS scan was determined automatically by the instrument software). Optimized MS parameters: spray voltage 3.00 kV, capillary temperature 280 °C, sheath gas rate 40.0 units N_2_ (ca. 400 mL/min), auxiliary gas rate 1.0 units N_2_ (ca. 10 mL/min). Source fragmentation with a mild potential (35 V) was turned on to avoid the formation of cluster ions, while producing no actual fragmentation.

MS/MS spectra were obtained in the data-dependent acquisition (DDA) mode, in which the three most intense ions in the full-scan mass spectrum are subjected to MS/MS analysis. The MS/MS spectra of the selected ions were collected with collision induced dissociation (CID) fragmentation, wideband activation mode, isolation width ±2.00 Da, collision energy 45 units, activation Q 0.250 units, and activation time 80 ms. 

### 4.4. Molecular Networking

Raw data obtained from the LC-MS/MS run were preliminarily processed using the msconvert program from the ProteoWizard suite [22] to convert the files in the open data format mzXML. In addition, the program was set to remove ^13^C isotope peaks from the MS/MS spectra during the conversion process.

The molecular network was created using the online workflow at GNPS. The data was filtered by removing all MS/MS peaks within ±17 Da of the precursor *m*/*z*. MS/MS spectra were window filtered by choosing only the top six peaks in the ±50 Da window throughout the spectrum. The data was then clustered with MS-Cluster with a parent mass tolerance of 0.02 Da and a MS/MS fragment ion tolerance of 0.02 Da to create consensus spectra. Further, consensus spectra that contained less than four spectra were discarded. A network was then created where edges were filtered to have a cosine score above 0.5 and more than six matched peaks. Further edges between two nodes were kept in the network if and only if each of the nodes appeared in each other’s respective top 10 most similar nodes. Data were imported into Cytoscape 3.6.0 (available online: http://www.cytoscape.org/) and displayed as a network of nodes and edges.

### 4.5. In Vitro Cytotoxic Activity

In vitro cytotoxicity of isolated compounds against HeLa cells was evaluated using the MTT [3-(4,5-dimethyl-2-thiazolyl)-2,5-diphenyl-2H-tetrazolium bromide] assay, using a procedure adapted from a previous study [31]. HeLa cells were obtained from the Pasteur Institute of Iran, Tehran. Cells were incubated in a humidified incubator with 5% CO_2_ at 37 °C and fed with Dulbecco’s Modified Eagle′s medium (DMEM). The medium was supplemented with 10% (*v*/*v*) FBS and penicillin (100 IU/mL), and streptomycin (100 µg/mL). A cell suspension (2 × 10^5^ cells/mL) was seeded in 96-well plates and incubated overnight to allow cell attachment. Compounds **1**–**4** were dissolved in dimethyl sulfoxide (DMSO) (the final concentration of DMSO in the plate was less than 1%) and 20 µL of different concentrations of samples were added and incubated for 72 h at 37 °C in a humidified atmosphere. Adriamycin was used as a positive control at 1 mg/mL, and 1% DMSO was used as negative control. Then cells were incubated with 20 µL of MTT solution (5 mg/mL) at 37 °C for 3 h. The medium was removed, DMSO (150 µL) was added to dissolve MTT-formazan crystals, and the absorbance was measured at 570 nm using a plate reader. The cell survival was calculated according to the following equation:

$$\%\;\mathrm{Cell}\;\mathrm{survival}=\frac{\mathrm{Absorbance}\;\mathrm{in}\;\mathrm{treated}\;\mathrm{wells}-\mathrm{Absorbance}\;\mathrm{in}\;\mathrm{blank}\;\mathrm{well}}{\mathrm{Absorbance}\;\mathrm{in}\;\mathrm{negative}\;\mathrm{control}\;\mathrm{or}\;\mathrm{untreated}\;\mathrm{well}-\mathrm{Absorbance}\;\mathrm{in}\;\mathrm{blank}\;\mathrm{well}}$$

IC_50_ were calculated using GraphPad Prism 6 (GraphPad Software, La Jolla, CA, USA) and are expressed as mean ± standard error (SE).

## 5. Conclusions

LC-MS/MS-based molecular networking was shown to be a powerful and unbiased method for a rapid profiling of saponins from holothurians. The method evidenced a clear difference between the saponin composition of a specimen of *H. atra* from the Persian Gulf and the previously reported results about specimens of *H. atra* of different geographical origin. The methodological improvement of MS/MS data acquisition and processing described in this paper for the analysis of *H. atra* will allow an easier application of molecular networking in future works on saponins. A new triterpene glycoside saponin, holothurin A5 (**1**), was isolated and characterized. The side chain of the new saponin **1**, unprecedented among triterpene glycosides, is characterized by an electrophilic enone function, which can undergo slow water or methanol addition under neutral conditions. Holothurin A5 (**1**) and the three known saponins **2**–**4** also isolated from *H. atra* were remarkably cytotoxic against HeLa cells, with IC_50_ values ranging from 1.2 to 2.5 µg/mL.

## Figures and Tables

**Table 1:** Putative identification of saponins present in the molecular network. MS/MS spectra of all compounds are reported in Figures S8–S21 (Supplementary Materials).

Measured *m*/*z*	Retention Time (min)	Molecular Formula	Putative Identification
859.38	23.8	C_41_H_63_O_17_S^−^	holothurin B
1165.51	23.1	C_54_H_85_O_25_S^−^	unknown
1167.53	23.6	C_54_H_87_O_25_S^−^	unknown
1181.51	23.2	C_54_H_85_O_26_S^−^	24-dehydroechinoside A (**4**)
1183.52	23.7	C_54_H_87_O_26_S^−^	echinoside A (**3**)
1195.48	21.5	C_54_H_83_O_27_S^−^	calcigeroside B
1197.50	21.8	C_54_H_85_O_27_S^−^	holothurin A (**2**)
1211.48	19.6	C_54_H_83_O_28_S^−^	unknown before this study (**1**)
1213.50	18.3	C_54_H_85_O_28_S^−^	holothurin D
1225.50	21.2	C_55_H_85_O_28_S^−^	unknown
1229.49	17.6, 18.1	C_54_H_85_O_29_S^−^	unknown before this study (**5**)
1243.51	19.5, 19.7	C_55_H_87_O_29_S^−^	unknown before this study (**6**)

**Table 2:** NMR Data of holothurin A5 (**1**) (^1^H 700 MHz, ^13^C 175 MHz, CD_3_OD).

Position	δ_C_, Type	δ_H_ (Mult, *J* in Hz)	Sugar	Position	δ_C_, Type	δ_H_ (Mult, *J* in Hz)
1	37.2 (CH_2_)	1.51 (m), 1.85 (m)	Xyl	1′	105.5 (CH)	4.43 (d, 7.5)
2	27.4 (CH_2_)	1.79 (m), 1.97 (m)		2′	82.2 (CH)	3.56 (dd, 8.9, 7.5)
3	90.0 (CH)	3.13 (br. d, 12.0)		3′	75.5 (CH)	3.73 (t, 8.9)
4	40.9 (C)			4′	77.1 (CH)	4.22 (m)
5	53.5 (CH)	0.98 (d, 12.4)		5′	64.0 (CH_2_)	3.37 (t, 10.1), 4.20 (m)
6	21.8 (CH_2_)	1.57 (m), 1.76 (m)				
7	28.8 (CH_2_)	1.46 (m), 1.77 (m)	Qui	1′′	104.9 (CH)	4.61 (d, 7.6)
8	41.6 (CH)	3.02 (dd, 4.0, 13.2)		2′′	76.2 (CH)	3.29 (dd, 9.0, 7.6)
9	156.2 (C)			3′′	75.6 (CH)	3.47 (t, 9.0)
10	40.7 (C)			4′′	86.5 (CH)	3.17 (t, 9.0)
11	115.2 (CH)	5.39 (br. d, 5.7)		5′′	72.2 (CH)	3.46 (m)
12	71.6 (CH)	4.55 (br. d, 5.7)		6′′	17.8 (CH_3_)	1.36 (d, 6.1)
13	60.0 (C)					
14	47.3 (C)		Glc	1′′′	104.4 (CH)	4.42 (d, 7.9)
15	37.2 (CH_2_)	1.21 (m), 1.79 (m)		2′′′	74.2 (CH)	3.41 (m)
16	39.2 (CH_2_)	1.90 (m), 2.16 (m)		3′′′	87.2 (CH)	3.57 (t, 8.9)
17	88.5 (C)			4′′′	69.5 (CH)	3.41 (m)
18	175.0 (C)			5′′′	77.4 (CH)	3.39 (m)
19	22.6 (CH_3_)	1.16 (s)		6′′′	62.3 (CH_2_)	3.67 (dd, 11.9, 5.7), 3.89 (dd, 11.9, 2.1)
20	93.0 (C)					
21	21.1 (CH_3_)	1.67 (s)	OMeGlc	1′′′′	104.9 (CH)	4.58 (d, 7.3)
22	198.3 (C)			2′′′′	75.1 (CH)	3.32 (m)
23	121.3 (CH)	6.85 (d, 15.9)		3′′′′	87.3 (CH)	3.11 (t, 8.7)
24	157.6 (CH)	7.14 (d, 15.9)		4′′′′	70.8 (CH)	3.33 (m)
25	71.9 (C)			5′′′′	77.8 (CH)	3.33 (m)
26	28.8 (CH_3_)	1.34 (s)		6′′′′	62.5 (CH_2_)	3.64 (dd, 11.7, 5.7), 3.86 (dd, 11.7, 1.7)
27	28.8 (CH_3_)	1.34 (s)		OMe	60.8 (CH_3_)	3.63 (s)
30	16.9 (CH_3_)	0.92 (s)				
31	28.3 (CH_3_)	1.09 (s)				
32	19.8 (CH_3_)	1.29 (s)				

**Table 3:** NMR chemical shifts of the side chain of the two epimers of compounds **5** and **6** (^1^H 700 MHz, ^13^C 175 MHz, CD_3_OD).

Position	Proton Count	Compound 5				Compound 6			
		5a		5b		6a		6b	
		δ_C_	δ_H_	δ_C_	δ_H_	δ_C_	δ_H_	δ_C_	δ_H_
17	C	88.1	-	88.4	-	88.1		88.4	
18	C	175.0	-	175.0	-	175.0		175.0	
19	CH_3_	22.6	1.16	22.6	1.16	22.6	1.16	22.6	1.16
20	C	93.0		93.0		92.6		93.2	
21	CH_3_	21.1	1.674	21.1	1.667	21.1	1.672	21.1	1.669
22	C	209.8		209.7		209.1		209.7	
23	CH_2_	41.8	2.83, 2.86	41.8	2.83, 2.86	41.8	2.88, 2.92	41.8	2.88, 2.92
24	CH	73.9	3.95	74.2	3.95	84.4	3.64	84.0	3.64
25	C	73.1		73.2		73.5		73.6	
26	CH_3_	24.1	1.152	24.2	1.156	24.6	1.136	24.8	1.144
27	CH_3_	26.8	1.218	26.7	1.208	26.8	1.220	26.7	1.210
OMe						61.3	3.46	61.3	3.46

**Table 4:** Cytotoxic activity of compounds **1**–**4** against HeLa cancer cells.

Compound	IC_50_ ± SE (µg/mL) ^a^
holothurin A5 (**1**)	1.9 ± 0.1
holothurin A (**2**)	1.4 ± 0.1
echinoside A (**3**)	1.2 ± 0.2
24-dehydroechinoside A (**4**)	2.5 ± 0.4
adriamycin	0.6 ± 0.1

^a^ Concentration (±standard error) that inhibited 50% of the growth of the HeLa human cervix carcinoma cell-line.

## Supplementary Materials

The following are available online at http://www.mdpi.com/1660-3397/17/2/86/s1, Tables S1–S3: ^1^H and ^13^C NMR data of compounds **2**–**4**; Figures S1–S5: ^1^H-NMR, COSY, HSQC, and HMBC spectra of compound **1** (700 MHz, CD_3_OD); Figures S6 and S7: ^1^H-NMR spectra of compounds **5** and **6** (700 MHz, CD_3_OD). Figures S8–S21: MS/MS spectra of compounds reported in Table 1.
