# Supplementary material for: Molecular Networking-Based Analysis of Cytotoxic Saponins from Sea Cucumber Holothuria atra
Source: Mar Drugs. 2019 Feb 1;17(2):86. doi: 10.3390/md17020086 (PMC6410324; doi:10.3390/md17020086)
Supplement: Supplementary file 1 [file marinedrugs-17-00086-s001.pdf]

Supplementary Materials

Table S1. ^{13}H and ^{13}C NMR data of holothurin A (**2**) in CD_3OD
Table S2. ^{13}H and ^{13}C NMR data of echinoside A (**3**) in CD_3OD
Table S3. ^{13}H and ^{13}C NMR data of 24-dehydroechinoside A (**4**) in CD_3OD
Figure S1. ^{1}H-NMR spectrum of holothurin A5 (**1**) (700 MHz, CD_3OD)
Figure S2. COSY spectrum of holothurin A5 (**1**) (700 MHz, CD_3OD)
Figure S3. HSQC spectrum of holothurin A5 (**1**) (700 MHz, CD_3OD, high field region)
Figure S4. HSQC spectrum of holothurin A5 (**1**) (700 MHz, CD_3OD, low field region)
Figure S5. HMBC spectrum of holothurin A5 (**1**) (700 MHz, CD_3OD)
Figure S6. ^{1}H-NMR spectrum of compound **5** (700 MHz, CD_3OD)
Figure S7. ^{1}H-NMR spectrum of compound **6** (700 MHz, CD_3OD)
Figure S8. MS/MS spectrum for node at *m/z* **859.38** (t_R= 23.8 min)
Figure S9. MS/MS spectrum for node at *m/z* **1165.51** (t_R= 23.1 min)
Figure S10. MS/MS spectrum for node at *m/z* **1167.53** (t_R= 23.6 min)
Figure S11. MS/MS spectrum for node at *m/z* **1181.51** (t_R= 23.2 min, **3**)
Figure S12. MS/MS spectrum for node at *m/z* **1183.52** (t_R= 23.7 min, **4**)
Figure S13. MS/MS spectrum for node at *m/z* **1195.48** (t_R= 21.5 min, **2**)
Figure S14. MS/MS spectrum for node at *m/z* **1197.50** (t_R= 21.8 min)
Figure S15. MS/MS spectrum for node at *m/z* **1211.48** (t_R= 19.6 min, **1**)
Figure S16. MS/MS spectrum for node at *m/z* **1213.50** (t_R= 18.3 min)
Figure S17. MS/MS spectrum for node at *m/z* **1225.50** (t_R= 21.2 min)
Figure S18. MS/MS spectrum for node at *m/z* **1229.49** (t_R= 17.6 min, **5a**)
Figure S19. MS/MS spectrum for node at *m/z* **1229.49** (t_R= 18.1 min, **5b**)
Figure S20. MS/MS spectrum for node at *m/z* **1243.51** (t_R= 19.5 min, **6a**)
Figure S21. MS/MS spectrum for node at *m/z* **1243.51** (t_R= 19.7 min, **6b**)

Table S1. ^{13}H and ^{13}C NMR data of holothurin A (**2**) in CD_3OD

Position	δ_C, type	δ_H (mult, *J* in Hz)	Sugar	Position	δ_C, type	δ_H (mult, *J* in Hz)
1	37.3 (CH_2)	1.51 (m), 1.84 (m)	Xyl	1'	105.5 (CH)	4.43 (d, 7.5)
2	27.4 (CH_2)	1.77 (m), 1.97 (m)		2'	82.2 (CH)	3.56 (dd, 8.9, 7.5)
3	90.0 (CH)	3.13 (br. d, 12.0)		3'	75.5 (CH)	3.73 (t, 8.9)
4	40.8 (C)			4'	77.1 (CH)	4.22 (m)
5	53.5 (CH)	0.97 (d, 12.4)		5'	64.0 (CH_2)	3.37 (t, 10.1), 4.20 (m)
6	21.8 (CH_2)	1.55 (m), 1.76 (m)				
7	28.8 (CH_2)	1.46 (m), 1.77 (m)	Qui	1''	104.9 (CH)	4.61 (d, 7.6)
8	41.6 (CH)	3.01 (dd, 4.0, 13.2)		2''	76.2 (CH)	3.29 (dd, 9.0, 7.6)
9	155.4 (C)			3''	75.6 (CH)	3.47 (t, 9.0)
10	40.7 (C)			4''	86.5 (CH)	3.17 (t, 9.0)
11	115.4 (CH)	5.36 (br. d, 5.7)		5''	72.2 (CH)	3.46 (m)
12	72.6 (CH)	4.53 (br. d, 5.7)		6''	17.8 (CH_3)	1.36 (d, 6.1)
13	60.1 (C)					
14	46.6 (C)		Glc	1'''	104.4 (CH)	4.42 (d, 7.9)
15	37.2 (CH_2)	1.16 (m), 1.79 (m)		2'''	74.2 (CH)	3.41 (m)
16	36.1 (CH_2)	2.07 (m), 2.53 (m)		3'''	87.2 (CH)	3.57 (t, 8.9)
17	88.2 (C)			4'''	69.5 (CH)	3.41 (m)
18	176.8 (C)			5'''	77.4 (CH)	3.39 (m)
19	22.7 (CH_3)	1.14 (s)		6'''	62.3 (CH_2)	3.67 (dd, 11.9, 5.7), 3.89 (dd, 11.9, 2.1)
20	90.2 (C)					
21	18.8 (CH_3)	1.49 (s)	OMeGlc	1''''	105.0 (CH)	4.58 (d, 7.3)
22	81.5 (C)			2''''	75.1 (CH)	3.32 (m)
23	26.8 (CH_2)	1.53 (m)		3''''	87.3 (CH)	3.11 (t, 8.7)
24	39.3 (CH_2)	1.78 (m)		4''''	70.8 (CH)	3.33 (m)
25	82.9 (C)			5''''	77.8 (CH)	3.33 (m)
26	28.9 (CH_3)	1.32 (s)		6''''	62.5 (CH_2)	3.64 (dd, 11.7, 5.7), 3.86 (dd, 11.7, 1.7)
27	27.7 (CH_3)	1.26 (s)		OMe	60.8 (CH_3)	3.64 (s)
30	16.9 (CH_3)	0.91 (s)				
31	28.3 (CH_3)	1.06 (s)				
32	20.0 (CH_3)	1.29 (s)				

Table S2. ^{13}H and ^{13}C NMR data of echinoside A (**3**) in CD_3OD

Position	δ_C, type	δ_H (mult, *J* in Hz)	Sugar	Position	δ_C, type	δ_H (mult, *J* in Hz)
1	37.3 (CH_2)	1.51 (m), 1.84 (m)	Xyl	1'	105.5 (CH)	4.43 (d, 7.5)
2	27.4 (CH_2)	1.77 (m), 1.97 (m)		2'	82.2 (CH)	3.56 (dd, 8.9, 7.5)
3	90.0 (CH)	3.13 (br. d, 12.0)		3'	75.5 (CH)	3.73 (t, 8.9)
4	40.8 (C)			4'	77.1 (CH)	4.22 (m)
5	53.5 (CH)	0.97 (d, 12.4)		5'	64.0 (CH_2)	3.37 (t, 10.1), 4.20 (m)
6	21.8 (CH_2)	1.57 (m), 1.76 (m)				
7	28.8 (CH_2)	1.46 (m), 1.77 (m)	Qui	1''	104.9 (CH)	4.61 (d, 7.6)
8	41.6 (CH)	3.02 (dd, 4.0, 13.2)		2''	76.2 (CH)	3.29 (dd, 9.0, 7.6)
9	155.4 (C)			3''	75.6 (CH)	3.47 (t, 9.0)
10	40.7 (C)			4''	86.5 (CH)	3.17 (t, 9.0)
11	115.4 (CH)	5.36 (br. d, 5.7)		5''	72.2 (CH)	3.46 (m)
12	72.2 (CH)	4.53 (br. d, 5.7)		6''	17.8 (CH_3)	1.36 (d, 6.1)
13	60.1 (C)					
14	46.6 (C)		Glc	1'''	104.4 (CH)	4.41 (d, 7.9)
15	37.2 (CH_2)	1.17 (m), 1.79 (m)		2'''	74.2 (CH)	3.41 (m)
16	36.1 (CH_2)	2.07 (m), 2.53 (m)		3'''	87.2 (CH)	3.57 (t, 8.9)
17	88.2 (C)			4'''	69.5 (CH)	3.41 (m)
18	176.8 (C)			5'''	77.4 (CH)	3.39 (m)
19	22.7 (CH_3)	1.14 (s)		6'''	62.3 (CH_2)	3.67 (dd, 11.9, 5.7), 3.89 (dd, 11.9, 2.1)
20	90.2 (C)					
21	22.7 (CH_3)	1.54 (s)	OMeGlc	1''''	105.0 (CH)	4.58 (d, 7.3)
22	39.5 (CH_2)	1.68 (m), 1.72 (m)		2''''	75.1 (CH)	3.32 (m)
23	23.1 (CH_2)	1.38 (m), 1.48 (m)		3''''	87.3 (CH)	3.11 (t, 8.7)
24	40.9 (CH_2)	1.21(m)		4''''	70.8 (CH)	3.34 (m)
25	29.1 (CH_2)	1.56 (m)		5''''	77.8 (CH)	3.32 (m)
26	23.1 (CH_3)	0.92 (d)		6''''	62.5 (CH_2)	3.63 (dd, 11.7, 5.7), 3.87 (dd, 11.7, 1.7)
27	22.9 (CH_3)	0.92 (d)		OMe	60.8 (CH_3)	3.64 (s)
30	16.9 (CH_3)	0.91 (s)				
31	28.3 (CH_3)	1.06 (s)				
32	20.0 (CH_3)	1.29 (s)				

Table S3. ^{13}H and ^{13}C NMR data of 24-dehydroechinoside A (**4**) in CD_3OD

Position	δC, type	δH (mult, *J* in Hz)	Sugar	Position	δC, type	δH (mult, *J* in Hz)
1	37.3 (CH_2)	1.51 (m), 1.84 (m)	Xyl	1'	105.5 (CH)	4.43 (d, 7.5)
2	27.4 (CH_2)	1.77 (m), 1.97 (m)		2'	82.2 (CH)	3.56 (dd, 8.9, 7.5)
3	90.0 (CH)	3.13 (br. d, 12.0)		3'	75.5 (CH)	3.73 (t, 8.9)
4	40.8 (C)			4'	77.1 (CH)	4.22 (m)
5	53.5 (CH)	0.97 (d, 12.4)		5'	64.0 (CH_2)	3.37 (t, 10.1), 4.20 (m)
6	21.8 (CH_2)	1.57 (m), 1.76 (m)				
7	28.8 (CH_2)	1.46 (m), 1.77 (m)	Qui	1''	104.9 (CH)	4.61 (d, 7.6)
8	41.6 (CH)	3.02 (dd, 4.0, 13.2)		2''	76.2 (CH)	3.29 (dd, 9.0, 7.6)
9	155.4 (C)			3''	75.6 (CH)	3.47 (t, 9.0)
10	40.7 (C)			4''	86.5 (CH)	3.17 (t, 9.0)
11	115.4 (CH)	5.36 (br. d, 5.7)		5''	72.2 (CH)	3.46 (m)
12	72.2 (CH)	4.53 (br. d, 5.7)		6''	17.8 (CH_3)	1.36 (d, 6.1)
13	60.1 (C)					
14	46.6 (C)		Glc	1'''	104.4 (CH)	4.41 (d, 7.9)
15	37.2 (CH_2)	1.17 (m), 1.79 (m)		2'''	74.2 (CH)	3.41 (m)
16	36.1 (CH_2)	2.07 (m), 2.53 (m)		3'''	87.2 (CH)	3.57 (t, 8.9)
17	88.2 (C)			4'''	69.5 (CH)	3.41 (m)
18	176.8 (C)			5'''	77.4 (CH)	3.39 (m)
19	22.7 (CH_3)	1.14 (s)		6'''	62.3 (CH_2)	3.67 (dd, 11.9, 5.7), 3.89 (dd, 11.9, 2.1)
20	90.2 (C)					
21	22.7 (CH_3)	1.54 (s)	OMeGlc	1''''	105.0 (CH)	4.58 (d, 7.3)
22	39.1 (CH_2)	1.75 (m)		2''''	75.1 (CH)	3.32 (m)
23	23.7 (CH_2)	2.06 (m), 2.17 (m)		3''''	87.3 (CH)	3.11 (t, 8.7)
24	124.6 (CH)	5.14 (t, 6.7)		4''''	70.8 (CH)	3.34 (m)
25	133.0 (C)			5''''	77.8 (CH)	3.32 (m)
26	17.5 (CH_3)	1.64 (s)		6''''	62.5 (CH_2)	3.63 (dd, 11.7, 5.7), 3.87 (dd, 11.7, 1.7)
27	25.5 (CH_3)	1.69 (s)		OMe	60.8 (CH_3)	3.63 (s)
30	16.9 (CH_3)	0.91 (s)				
31	28.3 (CH_3)	1.08 (s)				
32	20.0 (CH_3)	1.29 (s)				

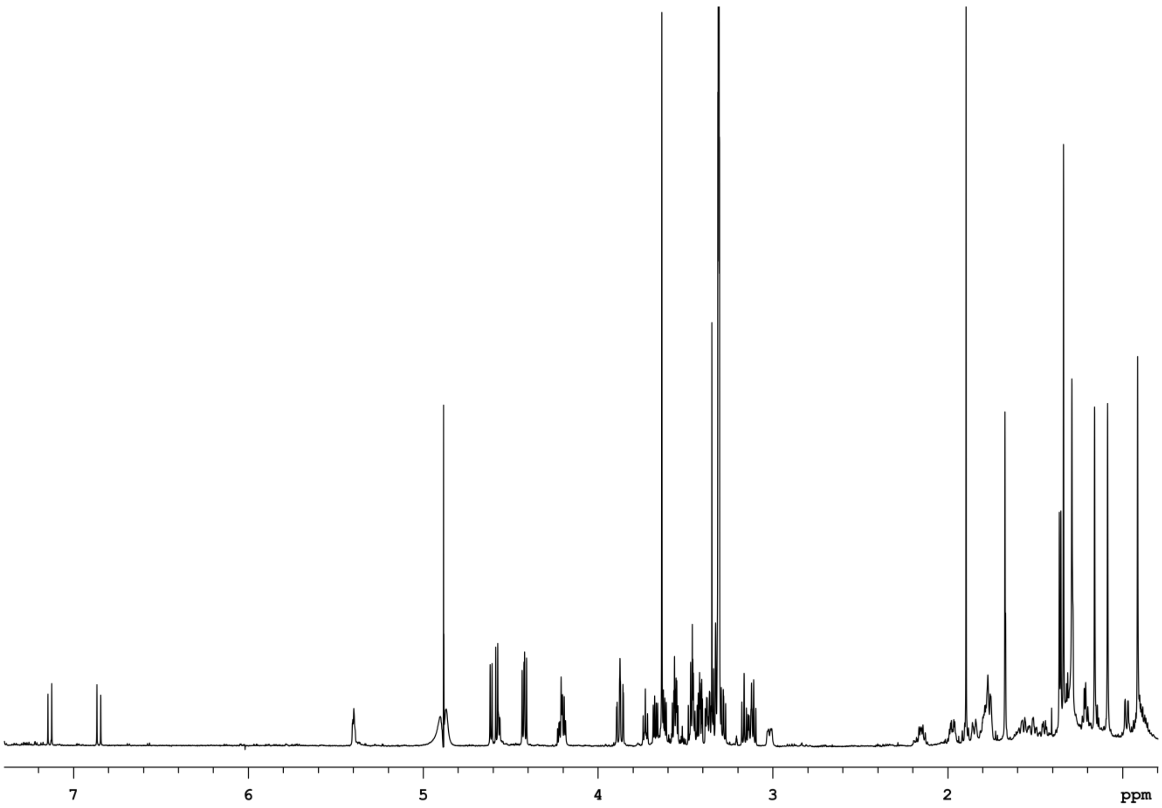

Figure S1. ^{1}H-NMR spectrum of holothurin A5 (**1**) (700 MHz, CD_3OD).

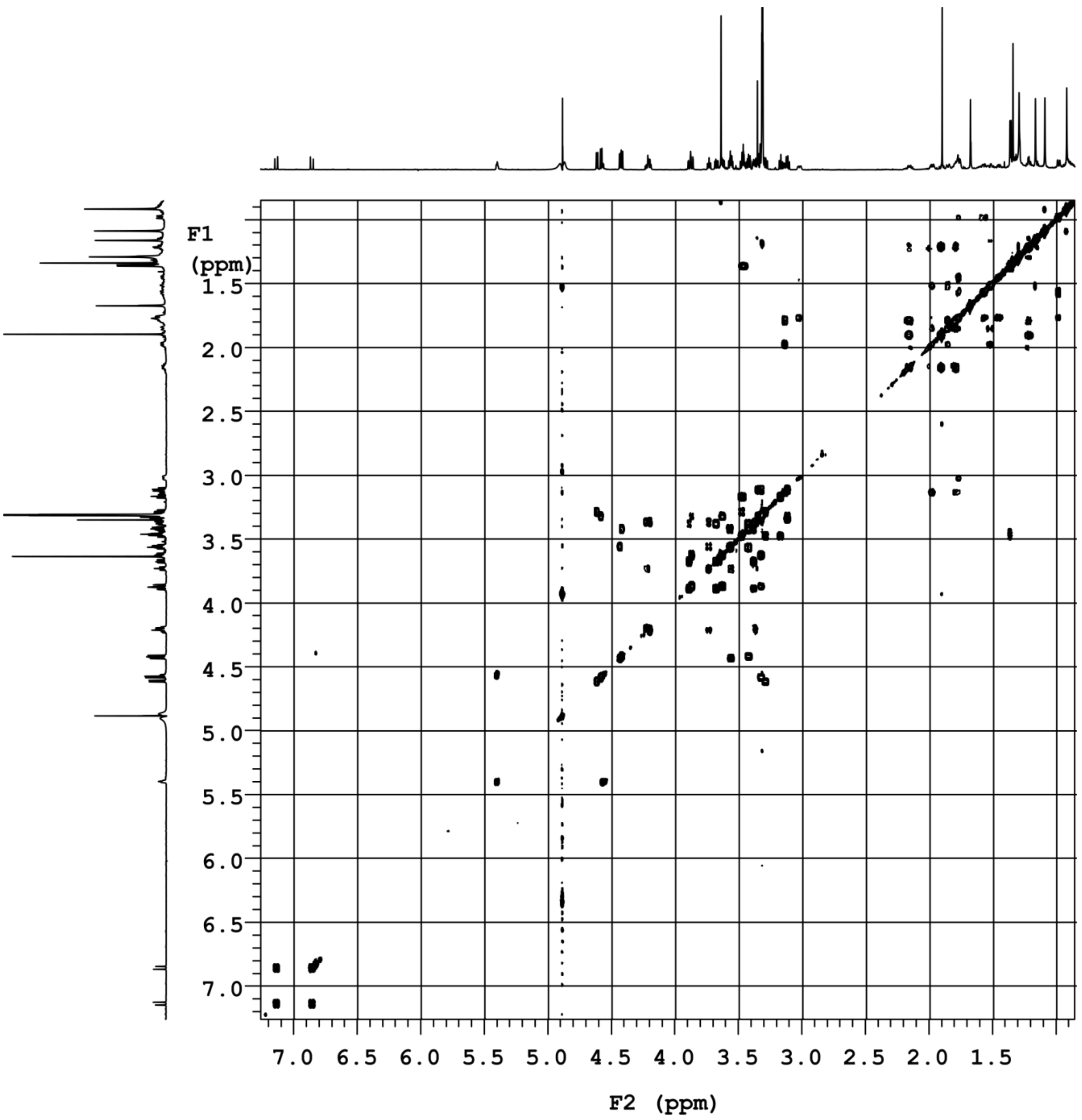

Figure S2. COSY spectrum of holothurin A5 (**1**) (700 MHz, CD_3OD)

.

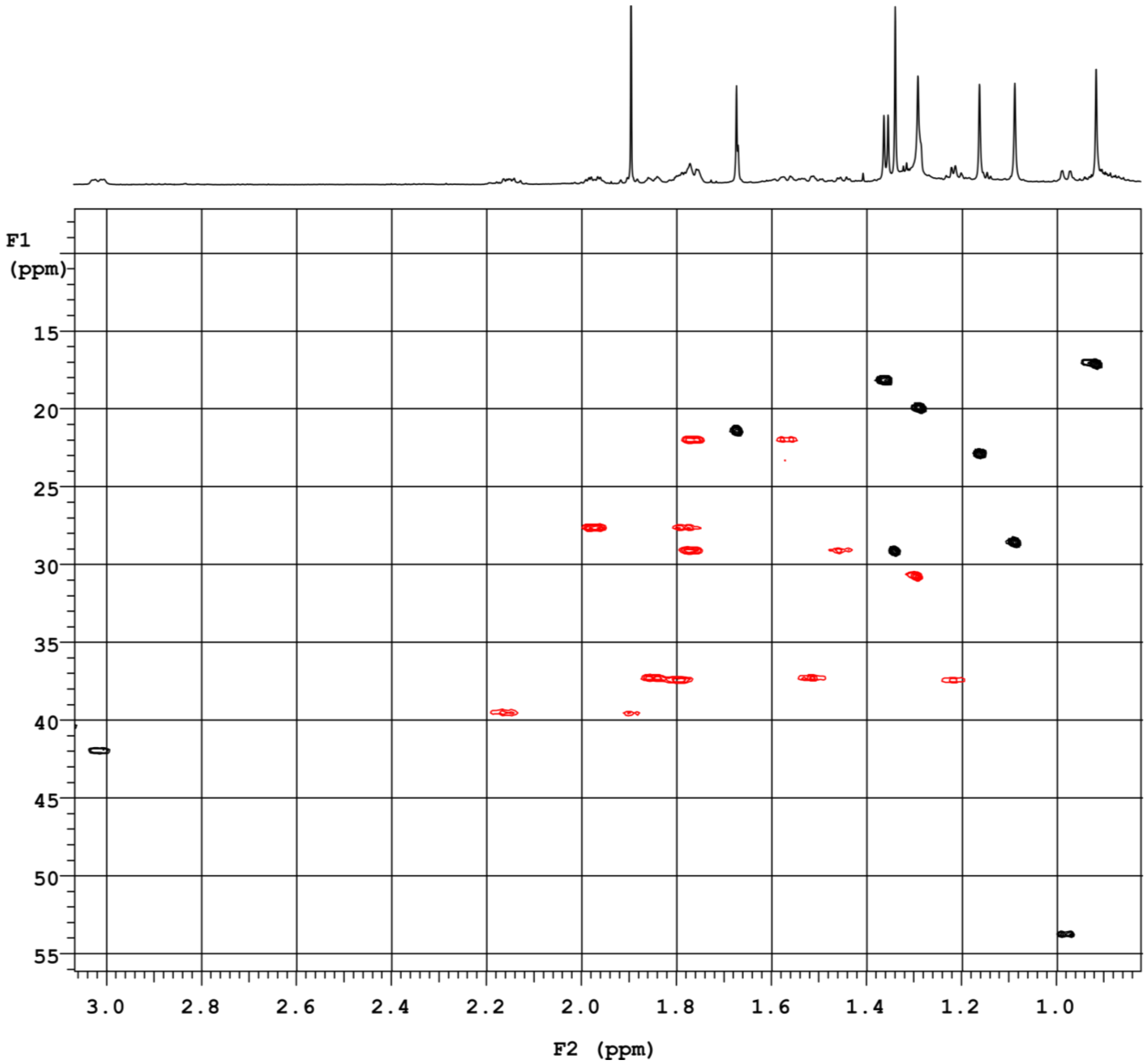

Figure S3. HSQC spectrum of holothurin A5 (**1**) (700 MHz, CD_3OD, high field region)

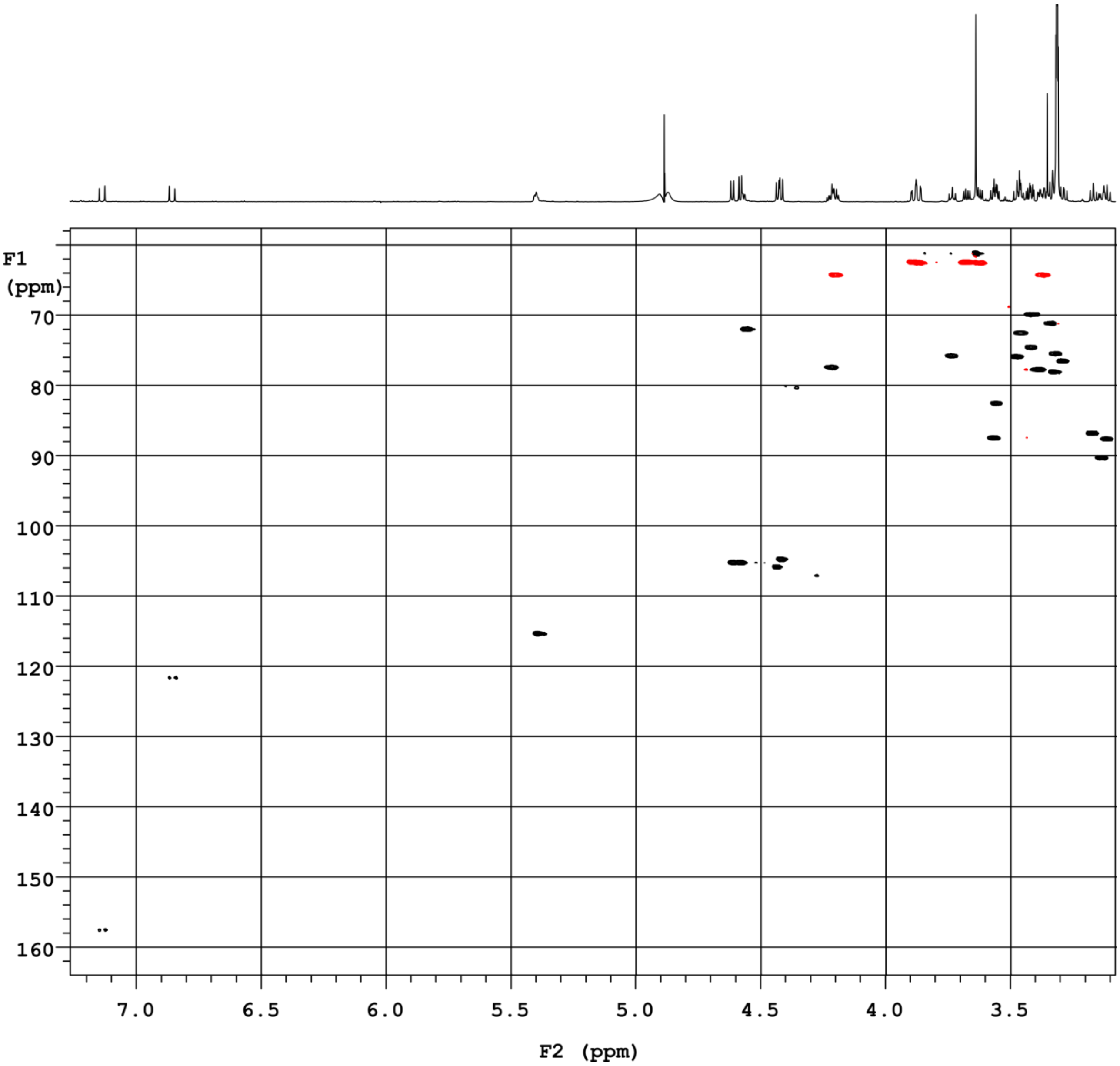

Figure S4. HSQC spectrum of holothurin A5 (**1**) (700 MHz, CD_3OD, low field region)

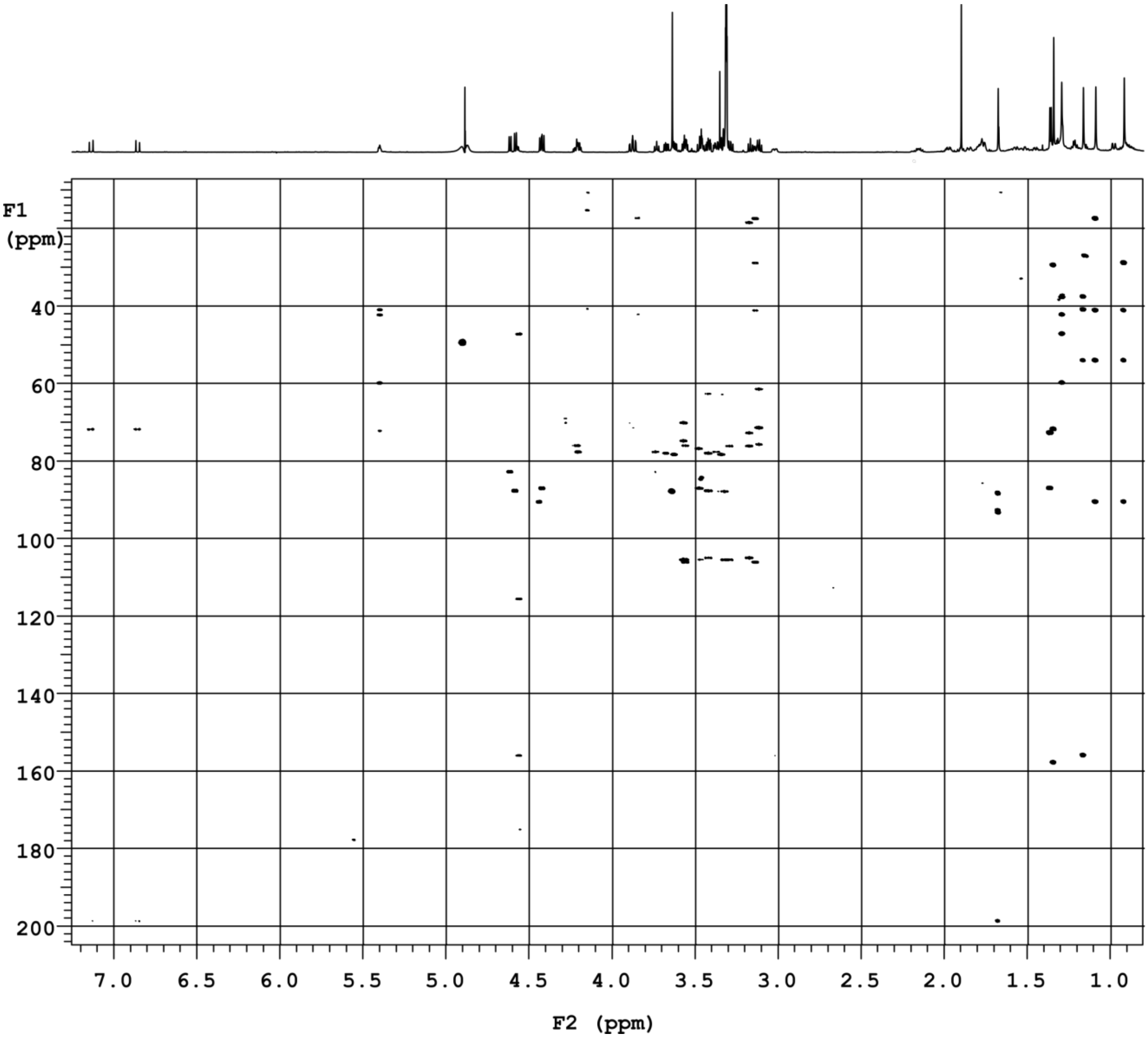

Figure S5. HMBC spectrum of holothurin A5 (**1**) (700 MHz, CD_3OD)

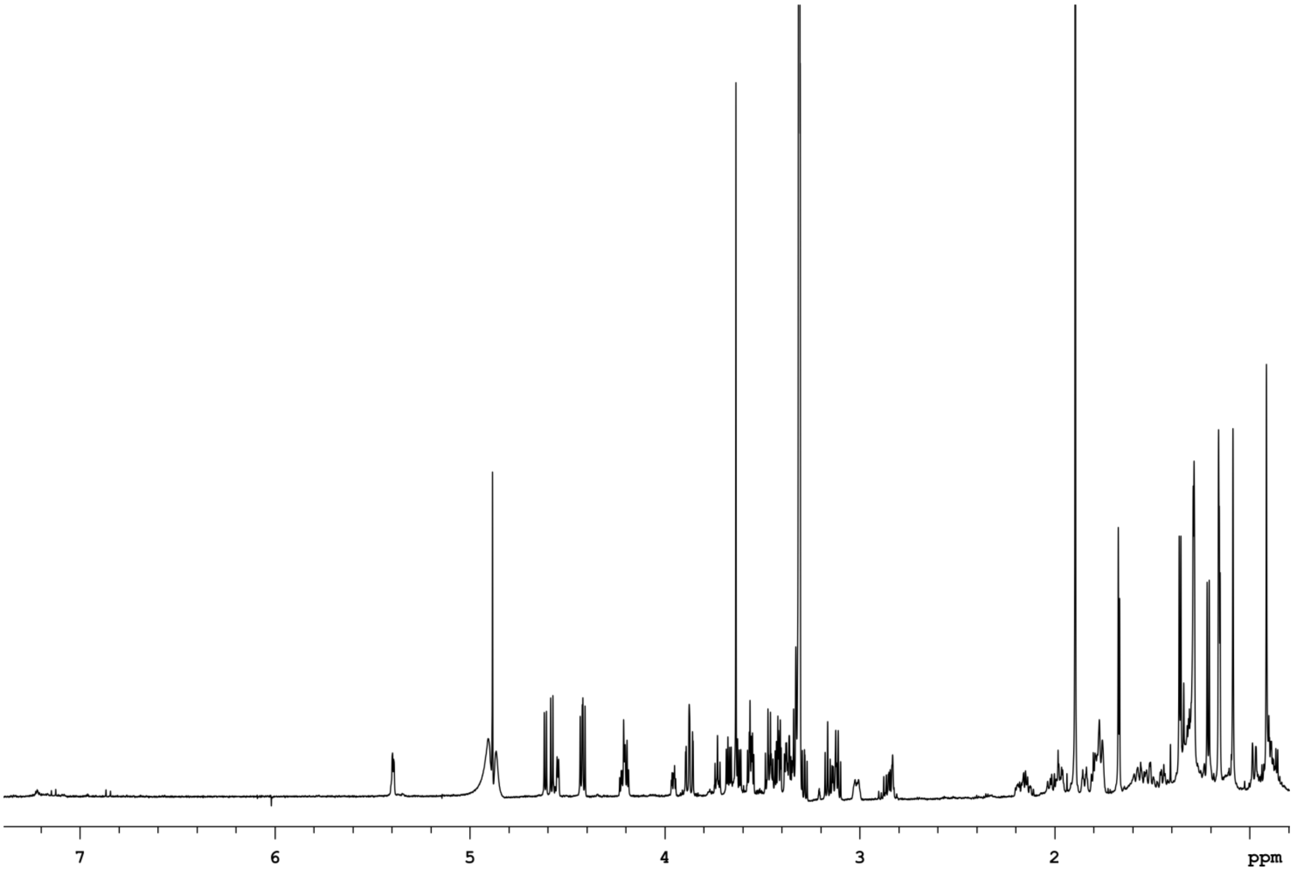

Figure S6. ^{1}H-NMR spectrum of compound **5** (700 MHz, CD_3OD).

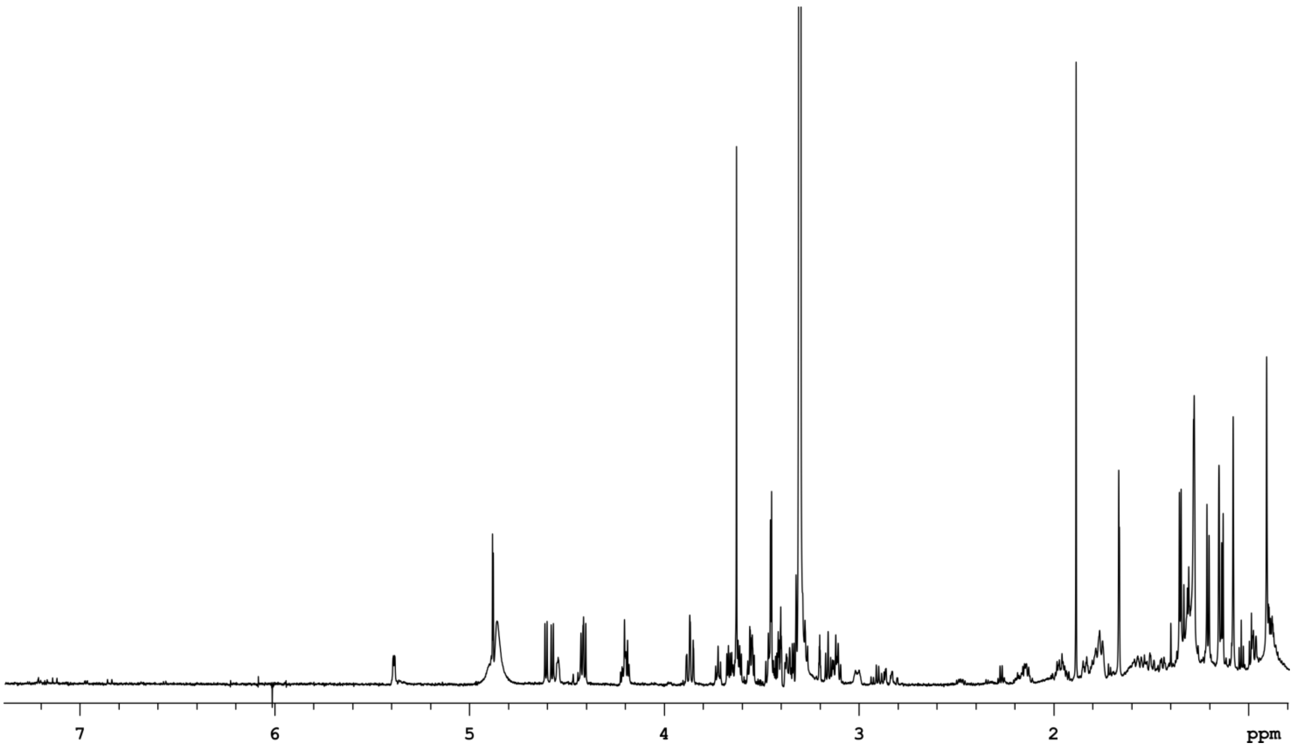

Figure S7. ^{1}H-NMR spectrum of compound **6** (700 MHz, CD_3OD).

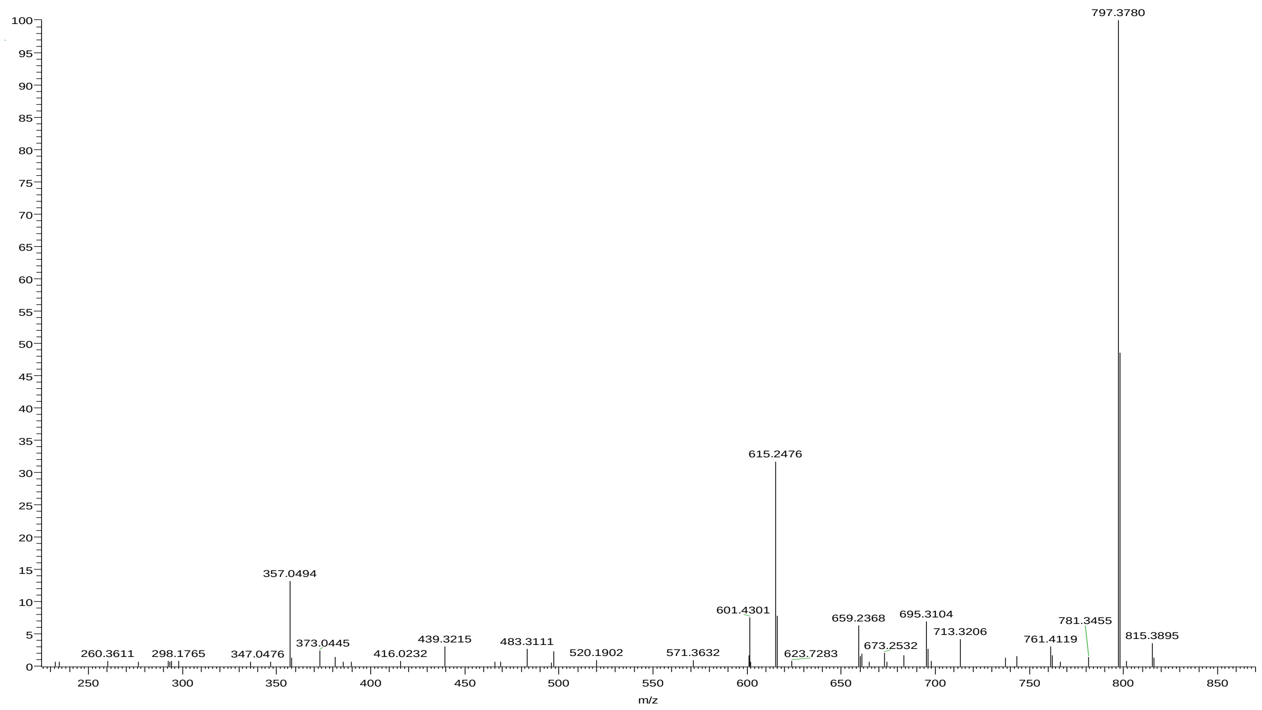

Figure S8. MS/MS spectrum for node at m/z 859.38 (t_R= 23.8 min)

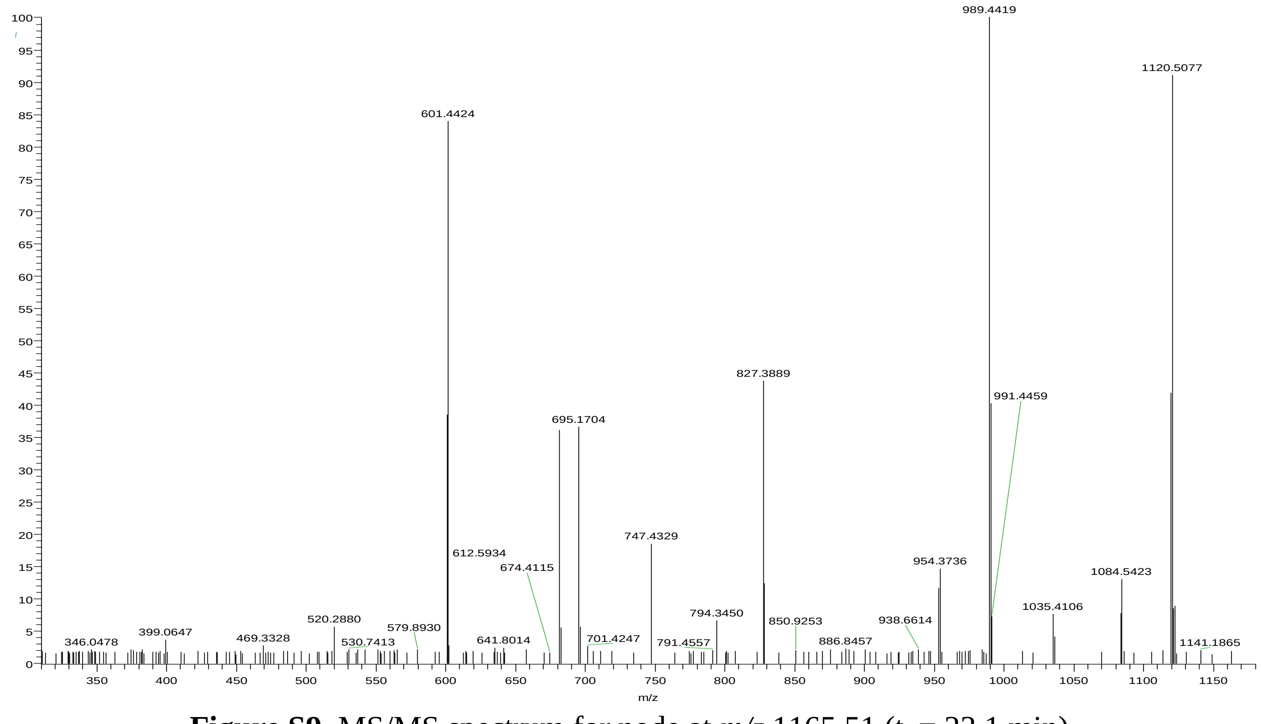

Figure S9. MS/MS spectrum for node at *m/z* 1165.51 (t_R= 23.1 min).

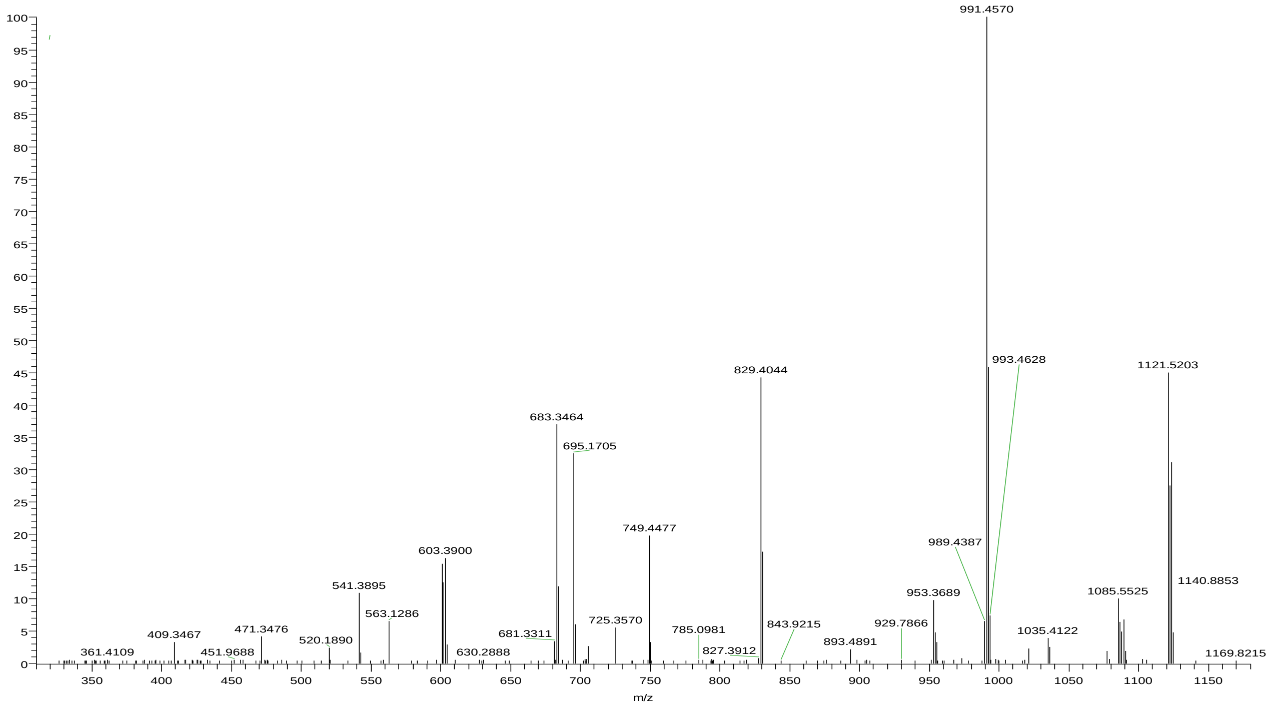

Figure S10. MS/MS spectrum for node at *m/z* 1167.53 (t_R= 23.6 min).

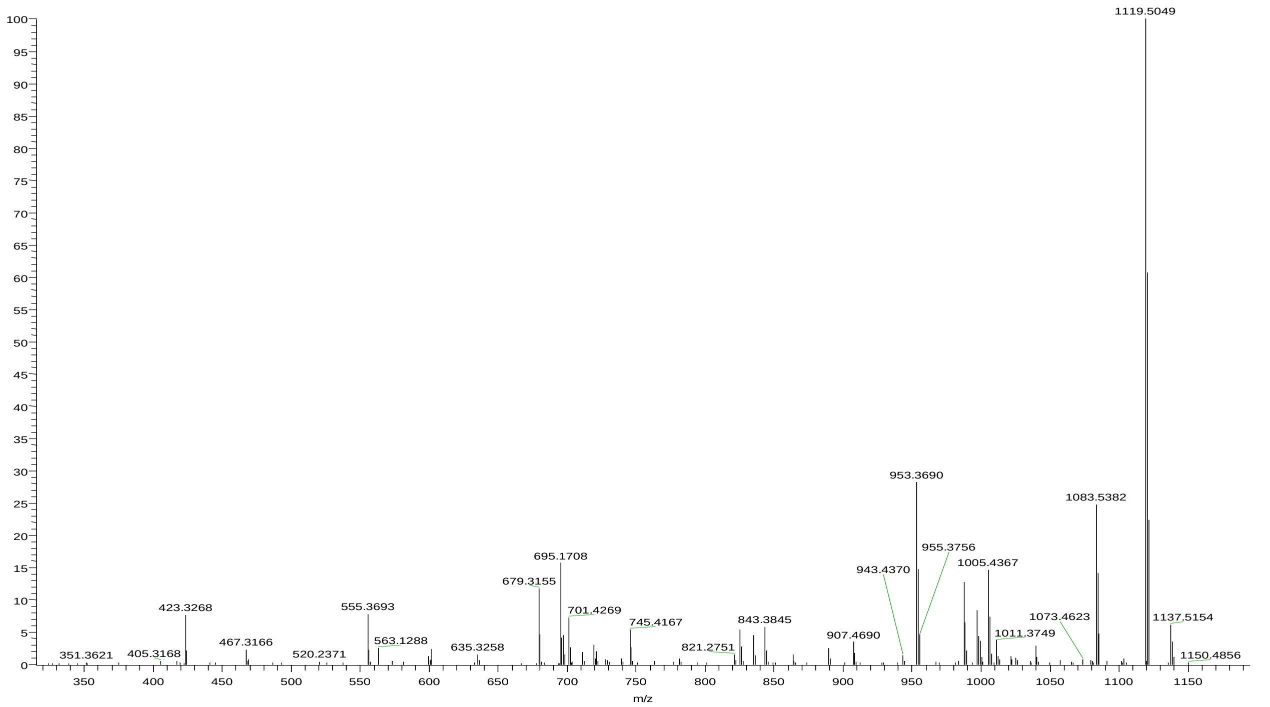

Figure S11. MS/MS spectrum for node at *m/z* 1181.51 (t_R= 23.2 min, compound **3**).

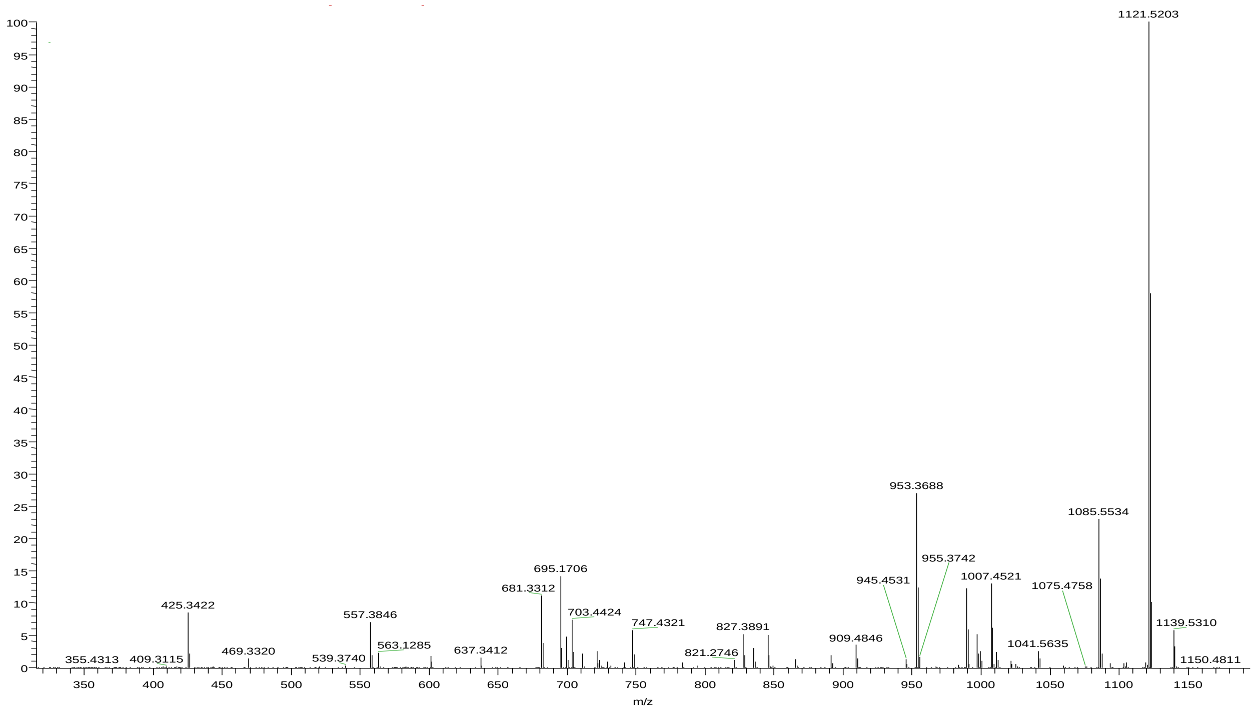

Figure S12. MS/MS spectrum for node at *m/z* 1183.52 (t_R= 23.7 min, compound **4**).

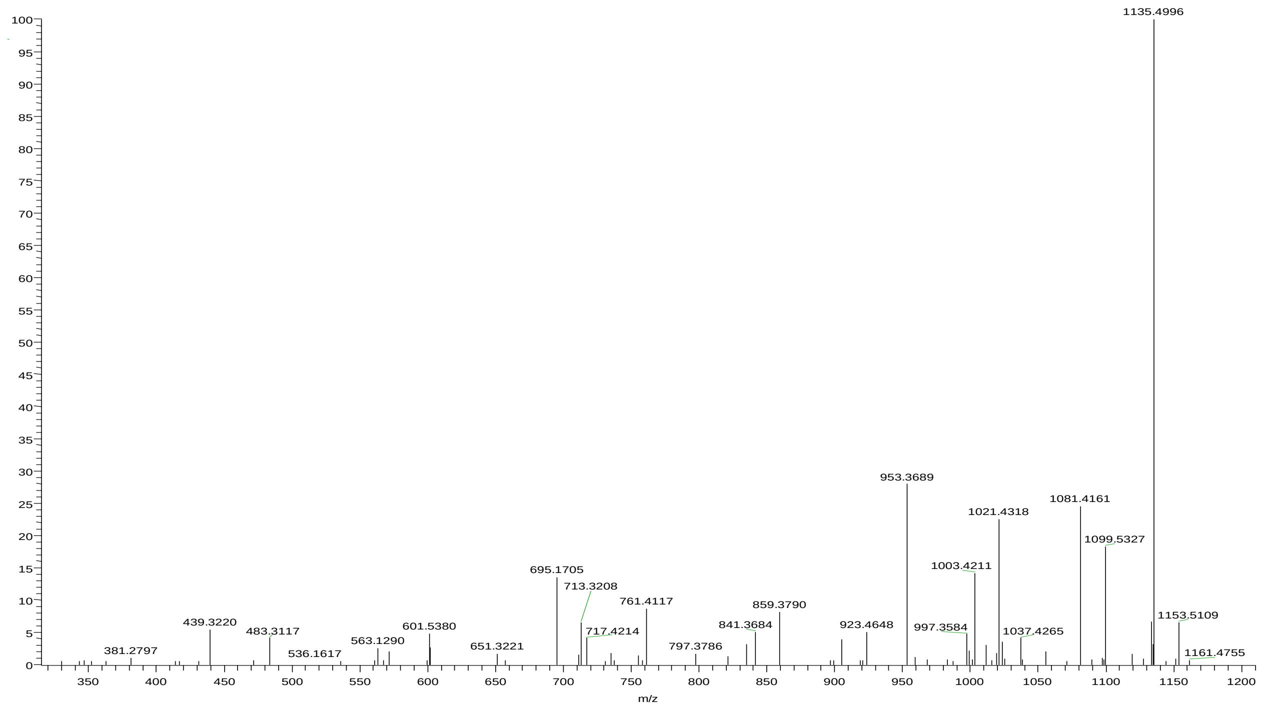

Figure S13. MS/MS spectrum for node at *m/z* 1195.48 (t_R= 21.5 min, compound **2**).

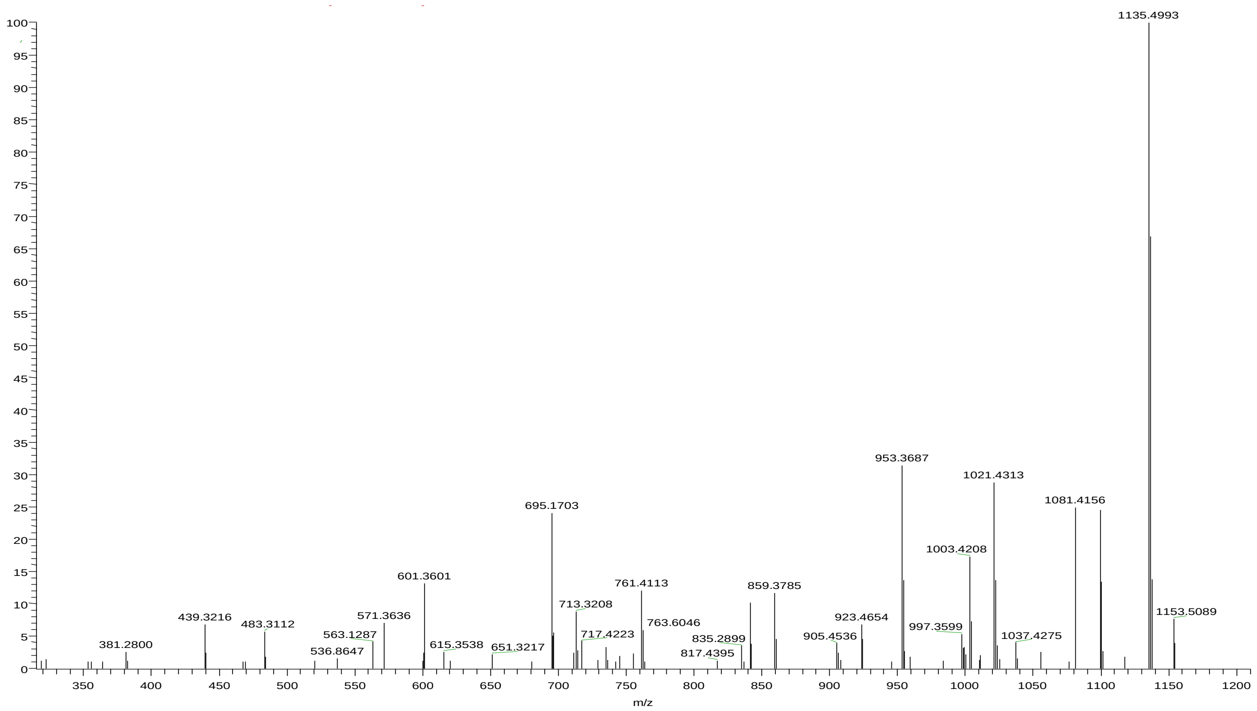

Figure S14. MS/MS spectrum for node at *m/z* 1197.50 (t_R= 21.8 min).

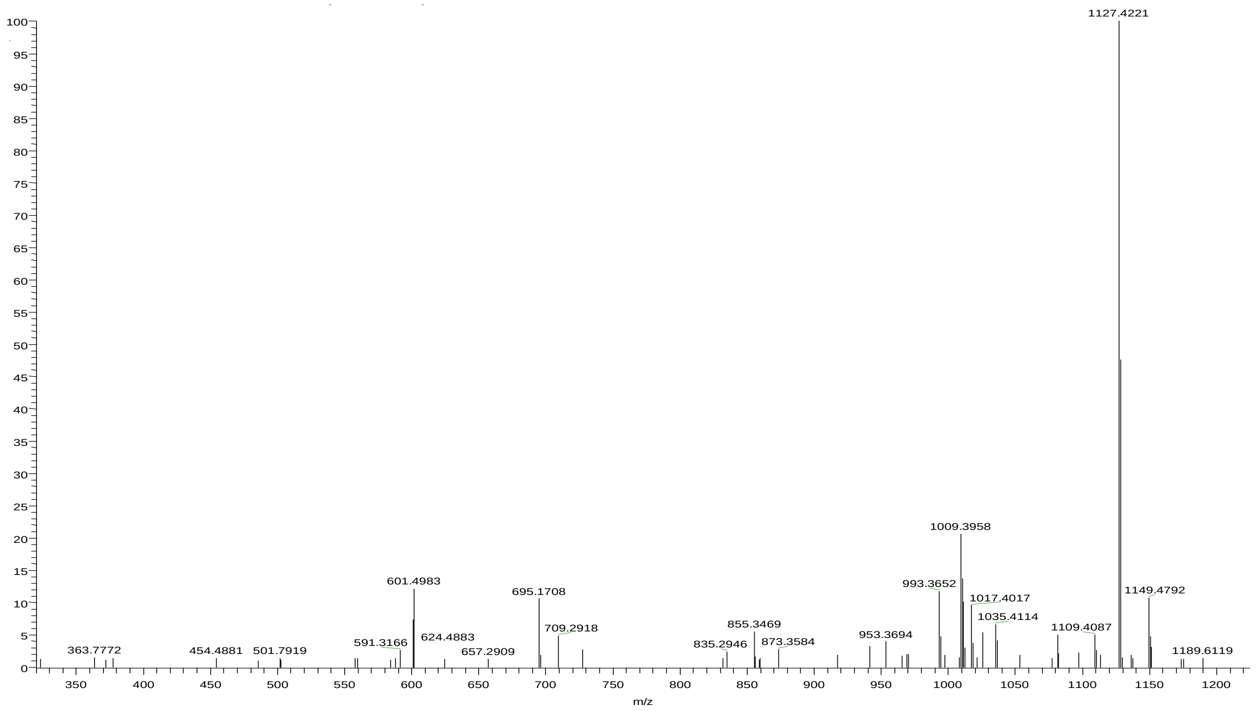

Figure S15. MS/MS spectrum for node at *m/z* 1211.48 (t_R= 19.6 min, compound **1**).

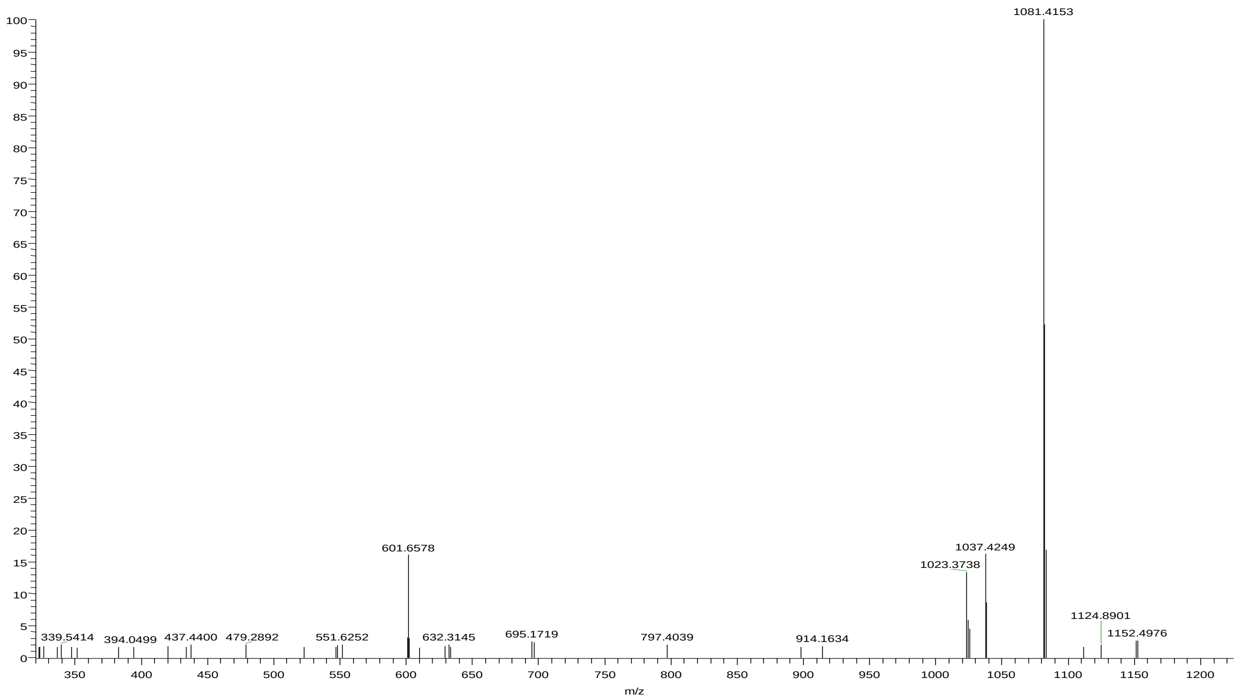

Figure S16. MS/MS spectrum for node at *m/z* 1213.50 (t_R= 18.3 min).

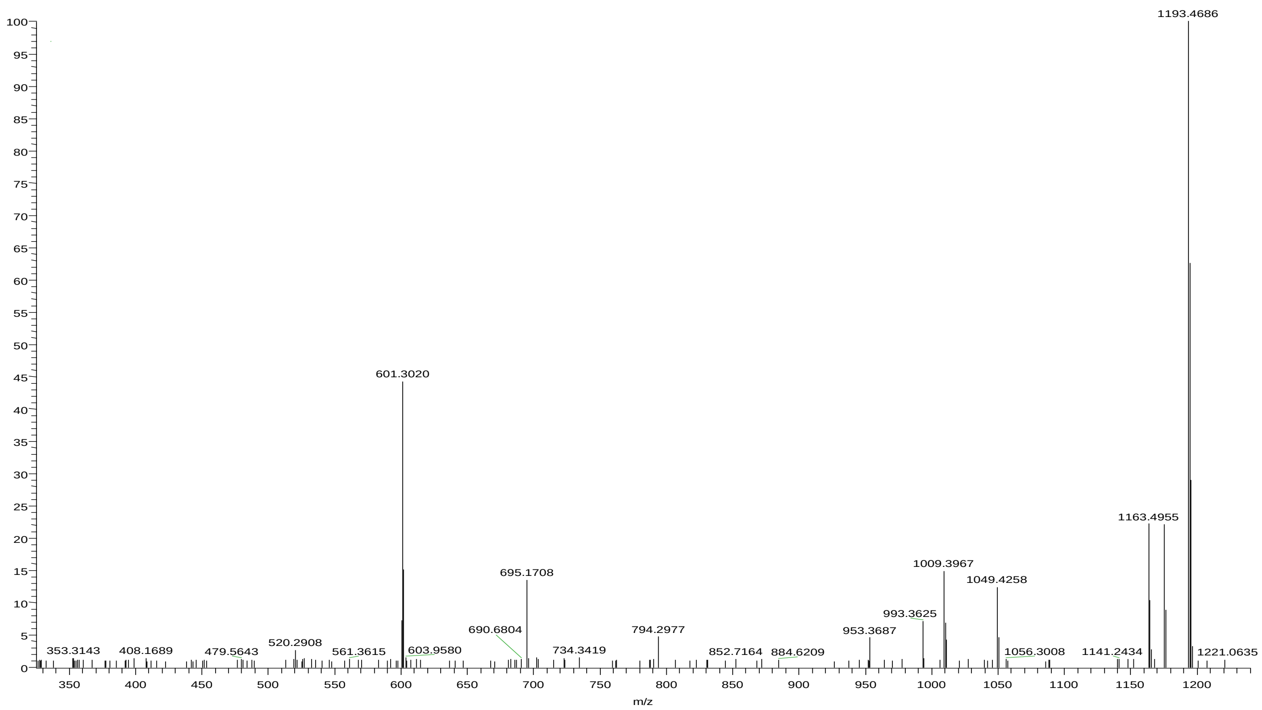

Figure S17. MS/MS spectrum for node at *m/z* 1225.50 (t_R= 21.2 min).

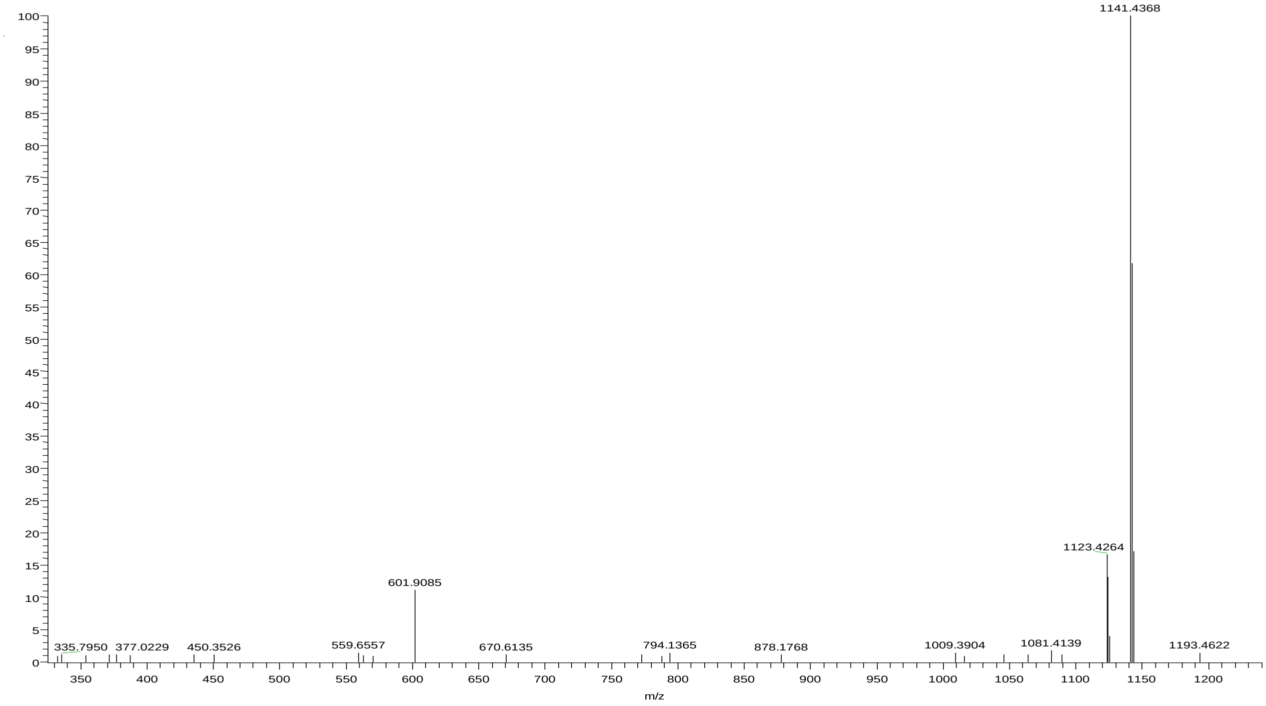

Figure S18. MS/MS spectrum for node at *m/z* 1229.49 (t_R= 17.6 min, compound **5a**).

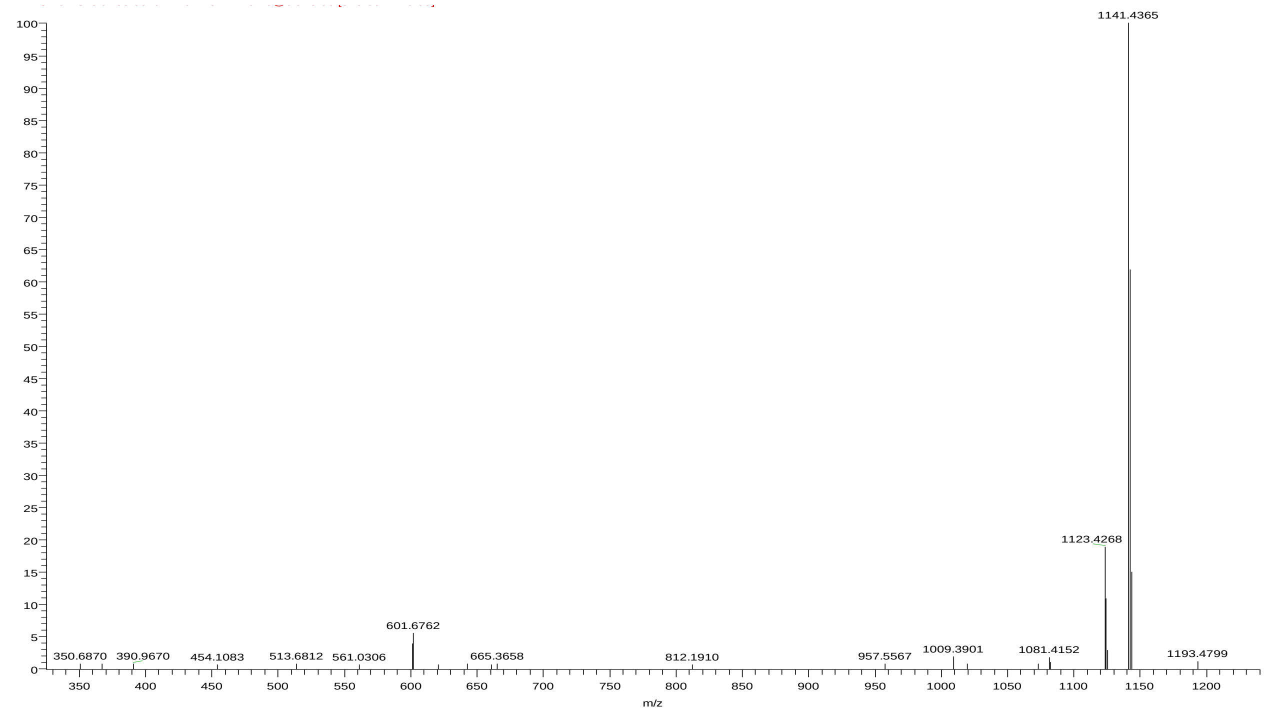

Figure S19. MS/MS spectrum for node at *m/z* 1229.49 (t_R= 18.1 min, compound **5b**).

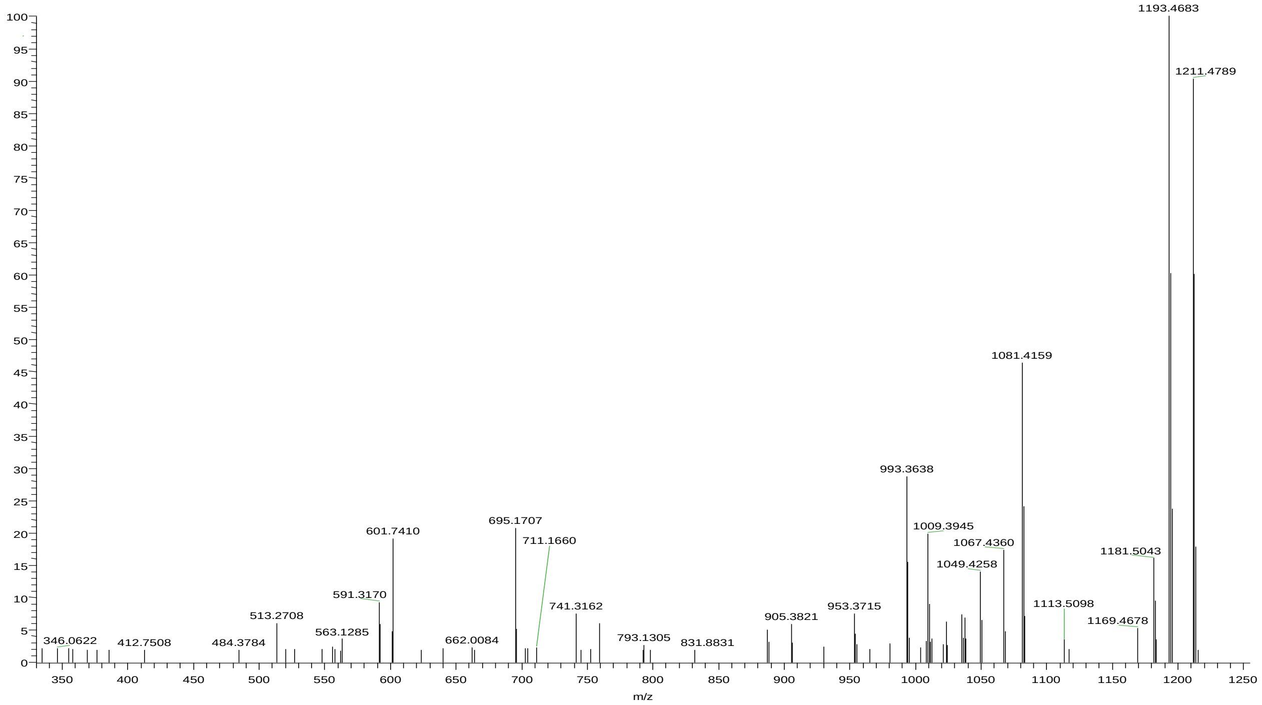

Figure S20. MS/MS spectrum for node at *m/z* 1243.51 (t_R= 19.5 min, compound **6a**).

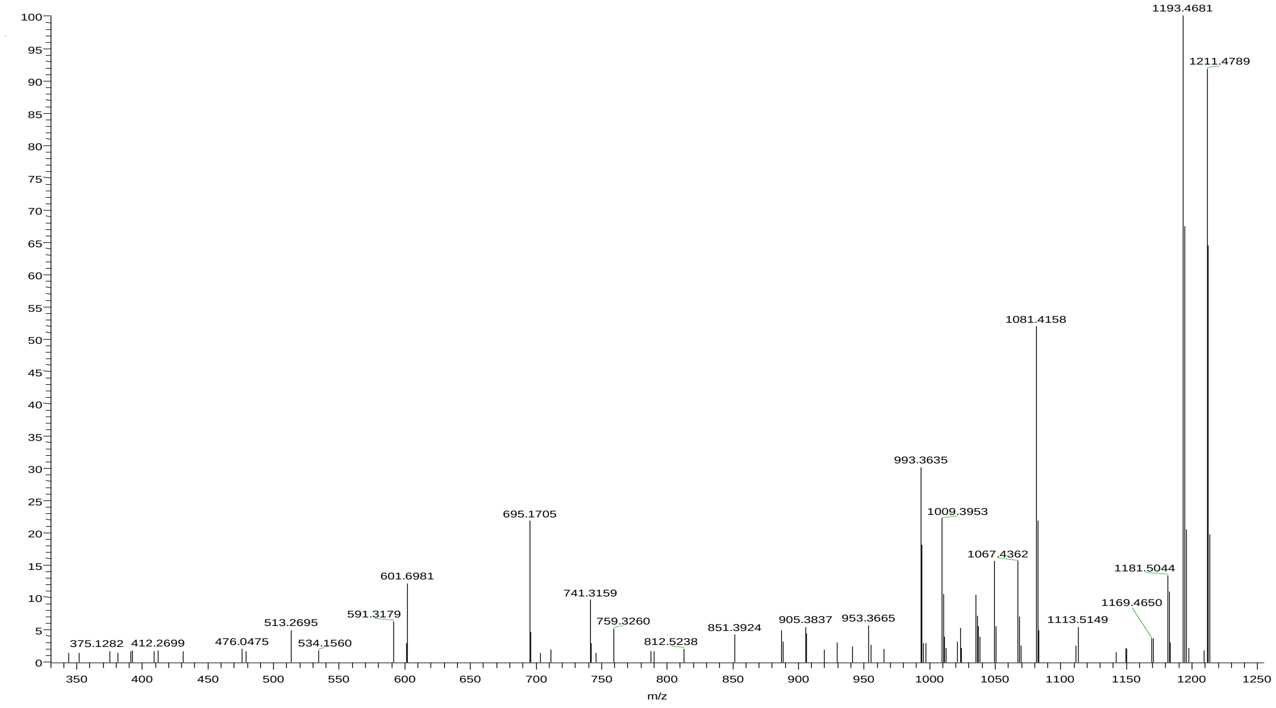

Figure S21. MS/MS spectrum for node at *m/z* 1243.51 (t_R= 19.7 min, compound **6b**).
